# Modified Rice Bran Arabinoxylan by *Lentinus edodes* Mycelial Enzyme as an Immunoceutical for Health and Aging—A Comprehensive Literature Review

**DOI:** 10.3390/molecules28176313

**Published:** 2023-08-29

**Authors:** Soo Liang Ooi, Peter S. Micalos, Sok Cheon Pak

**Affiliations:** 1 School of Dentistry and Medical Sciences, Charles Sturt University, Bathurst, NSW 2795, Australia; sooi@csu.edu.au; 2 School of Dentistry and Medical Sciences, Charles Sturt University, Port Macquarie, NSW 2444, Australia; pmicalos@csu.edu.au

**Keywords:** biobran, nutraceutical, anti-aging, MGN-3, natural products, natural killer cells, physiological activities

## Abstract

Rice bran arabinoxylan compound (RBAC) is derived from defatted rice bran enzymatically treated with *Lentinus edodes* mycelium. This review explores biologically active compounds and mechanisms of action that support RBAC as an immunomodulating nutraceutical in generally healthy and/or aging individuals. Thirty-seven (*n* = 37) primary research articles fulfilled the selection criteria for review. Most research is based on Biobran MGN-3, which consists of complex heteropolysaccharides with arabinoxylan as its primary structure while also containing galactan and glucan. RBAC was found to invoke immunological activities through direct absorption via the digestive tract and interaction with immune cells at the Peyer’s patches. RBAC was shown to promote innate defence by upregulating macrophage phagocytosis and enhancing natural killer cell activity while lowering oxidative stress. Through induction of dendritic cell maturation, RBAC also augments adaptive immunity by promoting T and B lymphocyte proliferation. RBAC acts as an immunomodulator by inhibiting mast cell degranulation during allergic reactions, attenuating inflammation, and downregulating angiogenesis by modulating cytokines and growth factors. RBAC has been shown to be a safe and effective nutraceutical for improving immune health, notably in aging individuals with reduced immune function. Human clinical trials with geriatric participants have demonstrated RBAC to have prophylactic benefits against viral infection and may improve their quality of life. Further research should explore RBAC’s bioavailability, pharmacodynamics, and pharmacokinetics of the complex heteropolysaccharides within. Translational research to assess RBAC as a nutraceutical for the aging population is still required, particularly in human studies with larger sample sizes and cohort studies with long follow-up periods.

## 1. Introduction

The immune response is the body’s ability to protect against infections by pathogens, such as harmful bacteria, fungi, parasites, and viruses, while stopping mutated host cells from becoming cancerous tumours [1,2]. These defensive mechanisms rely on the interrelationship of a complex network of organs (barriers and lymphoid system), cells (leukocytes) and proteins (cytokines and complement). The immune system comprises two arms that work closely together. Innate immunity is the first and is not specific to any antigens. The lines of defence include physical (skin and mucous membrane) and physiological (temperature, low pH and chemical mediators) barriers, plus cellular and inflammatory responses. The second arm is adaptive or antigen-specific immunity which recognises and memorises the initial exposure to an antigen and can mount a rapid and efficient response upon subsequent encounter [3].

Many factors can affect the optimal functioning of the human immune system, such as age, genetics, comorbidities, nutritional status, behaviour (e.g., smoking) and environment (e.g., chemical exposure) [4]. Aging, in particular, is known to cause physiological changes that reduce immune function, formally known as immunosenescence, and contribute to susceptibility to infectious diseases, chronic inflammation, and neurodegenerative diseases [5]. Not surprisingly, aging is associated with increasing healthcare demands, and with aging demographics, many countries face escalating healthcare costs [6]. Preventive care with early detection and immunomodulatory interventions through pharmaceuticals, vaccines, lifestyle changes, nutritional support, and dietary supplementations are strategies to reduce age-associated immune decline and the economic burdens of healthcare [7,8]. There has also been a growing interest in anti-aging products derived from food and natural sources, such as vitamins, carotenoids, flavonoids and minerals, as nutraceuticals to improve immunity and halt or even reverse the aging process [9].

Nutraceuticals are natural substances derived from edible sources that confer health benefits beyond their nutritional values [10]. Unlike synthetic molecules, which are typically flat and straightforward, naturally occurring compounds often contain enormously diverse and complex molecules [11]. They are structurally ‘optimised’ through evolutionary processes to serve specific biological functions, such as regulating the endogenous defence mechanisms against pathogenic organisms. As such, the complex molecules in natural products often enhance the immune response to infectious diseases and neoplasms [12].

In 2020, the global market size of all immune health nutraceuticals was valued at USD 55.3 billion [13]. The COVID-19 pandemic has further increased consumer interest in boosting immune health, and thus the market is expected to grow at a compound annual growth rate of 11.3% from 2021 to 2028 [13]. Evidently, there has been a heightening interest in using nutraceuticals to improve an individual’s immuno-competence for disease prevention and anti-aging, resulting in the creation of a new portmanteau term ‘immunoceuticals’, combining ‘immunity’ and ‘pharmaceutical’. Tieu et al. [14] categorised immunoceuticals as “any nutraceuticals demonstrating beneficial immunomodulatory mechanisms that support an optimal immune system and/or modify immunological status to defend against various diseases such as cancers and infectious or autoimmune diseases” (p. 2).

Rice bran arabinoxylan compound (RBAC) is a class of immunoceuticals defined as any heteropolysaccharide compound derived from hydrolysis of defatted rice bran enzymatically treated with *Lentinus edodes* mycelium [15]. Specifically, carbohydrase complexes from *L. edodes* mycelial are added during the processing to denature the complex cross-linked high molecular weight polysaccharides to improve bioavailability. The bioconversion produces partially hydrolysed rice bran hemicellulose with a high arabinoxylan content which is then heated, sterilised, and condensed to form the final product as a powder with high water-solubility [15]. Many authors have previously discussed RBAC’s production steps. Interested readers are referred to references [15,16,17].

The most well-known RBAC is Biobran MGN-3 from Daiwa Pharmaceutical Co., Ltd. (Tokyo, Japan; hereafter referred to as Daiwa), which is marketed worldwide as a nutraceutical under several different brand names such as Biobran, Ribraxx (in Australia), Lentin Plus (in Asia), or BRM4 (in the United States of America [USA]) [16]. Research in the immunological effects of RBAC, particularly Biobran MGN-3, has been ongoing for over 25 years, with research done in the USA, Japan, Republic of Korea, Egypt, Hungary, and many other countries [18]. Reported beneficial effects of RBAC include immunomodulation, synergistic anticancer properties, hepatoprotection, antiinflammation, antioxidation, and the potential to act on the psycho-neuro-immune axis. RBAC, most notably Biobran MGN-3, has been studied for potential applications in many conditions, including cancer, liver diseases, HIV, allergy, chronic fatigue, gastroenteritis, cold/flu, and diabetes [18].

The authors (SLO & SCP) have previously conducted reviews on RBAC. The first was a systematic evidence-based review of Biobran MGN-3 as a complementary therapy for conventional cancer treatment [19], followed by another narrative appraisal of RBAC’s potential clinical applications for cancer and beyond [15]. These reviews [15,19] focus primarily on the effects of RBAC as an intervention in disease conditions. However, being an immunoceutical available off the shelf, the primary use of RBAC is for prophylactic instead of curative. There is currently a lack of a comprehensive and critical assessment of the immunomodulating mechanisms of RBAC that support an optimal immune system for health and ageing, a gap in research that the present study aims to fill. Therefore, this review addresses the research question: ‘What are the biologically active compounds and mechanisms of action that support RBAC as an immunomodulating nutraceutical in generally healthy and/or aging individuals?’.

## 2. Materials and Methods

The present review was conducted on the heels of a scoping study that systematically searched, screened, assessed, extracted, and synthesised the available evidence sources from preclinical to clinical studies to map out the translational research of RBAC from inception until the end of 2022 [18]. The scoping study included all scholarly articles reporting results from primary research (basic, in vitro, animal, and human) on the effects of RBAC on any biological activities related to human health or disease conditions. Results of the scoping review covering all items of the PRISMA-ScR (Preferred Reporting Items for Systematic reviews and Meta-Analyses extension for Scoping Reviews) checklist were presented in a separate report [18]. Specifically, the scoping review included 98 articles published between 1998 to 2022. In addition, two recent RBAC articles published after the scoping study were also mentioned [20,21]. These articles formed the potential sources of evidence for the present review.

To answer the research question, the reviewers screened and shortlisted the articles (*n* = 100) found by the scoping study using the following concept-population-context criteria: (a) any studies on RBAC covering chemical analyses and biological activities (concept); (b) on healthy cells, tissues, animals or human participants including elderly (population); (c) concerning immune responses or its under- or overactivity (context). Furthermore, studies that utilised animal models induced with allergic reactions to study RBAC’s effects on hypersensitive immune responses were also included, as they are essential for understanding overactivity immunity. However, preclinical studies that investigated the impact of RBAC on the outcomes of therapeutic interventions for diseases utilising healthy cells, tissues, and animals were excluded.

Data and results from selected articles were extracted with specific details about the citation, study design, concept, context, methodology, outcome measures and key findings relevant to the research question. The evidence synthesis is illustrated graphically, diagrammatically, or in tabular form, with accompanying narrative summaries demonstrating how the results relate to the review question. All graphical illustrations were designed using Microsoft Publisher 365 (Redmond, WA, USA) with public domain clipart.

## 3. Results

Thirty-seven (*n* = 37) articles fulfilled the selection criteria and are included in the present analysis. The summary characteristics of the articles are available in Supplementary Table S1. Of these articles, 33 are journal articles, and the rest are short communication (1), thesis (1), and conference abstracts (2). Among them, 29 (78.4%) reported preclinical experiments, and 8 (21.6%) were human clinical studies. RBAC products from three companies were studied, namely Biobran MGN-3 from Daiwa, rice bran exo-biopolymer (RBEP) and Erom’s fermented rice bran (EFR) from Erom Co., Ltd. (Chuncheon, Republic of Korea; hereafter referred to as Erom), and bioprocessed polysaccharides (BPP), fermented black rice bran (CFP), or bioprocessed rice bran extract (BPRBE) from STR Biotech Co., Ltd. (Chuncheon, Republic of Korea; hereafter referred to as STR Biotech). Most articles are based on Biobran MGN-3 (*n* = 27, 73.0%), and 5 articles (13.5%) each are based on RBEP/EFR and STR Biotech’s RBAC derivatives.

### 3.1. Chemical Composition

Only one article by Miura et al. [22] attempted to identify the chemical structure of the active ingredient of RBAC responsible for its immunomodulatory activity. Through methanol fractional precipitation, the study discovered that several components of Biobran MGN-3 possess immunomodulatory activities. Among them are the 50% MeOH insoluble fraction (50 ppt) and subfractions II-4, II-5, and II-6 of 80% MeOH insoluble material eluted with 0.2 M NaCl which demonstrated macrophage-stimulating activity with increased nitric oxide (NO) formation while inducing interleukin (IL)-1β and tumour necrosis factor (TNF)-α production in vitro. Among these components, subfraction II-6 demonstrated the highest macrophage-stimulating activity and was found to have a molecular weight between 10,000–20,000 daltons.

**Figure 1:**
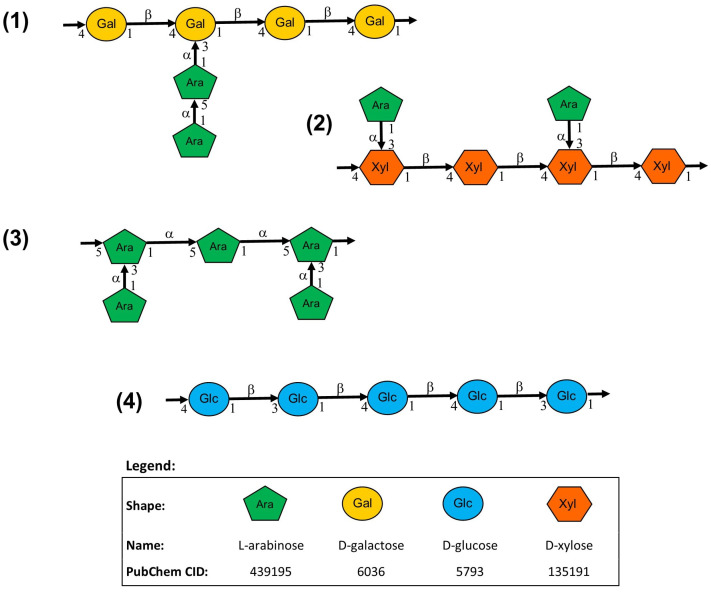
Four possible structures of the polysaccharides present in the most active fraction of Biobran MGN-3.

Through methylation analysis based on the sugar linkage composition of subfraction II-6, Miura et al. [22] proposed four possible polysaccharide structures in the active ingredient of Biobran MGN-3 to be: (1) arabinogalactan with a main chain of 1,4-β-galactan and side chains of arabinose, (2) arabinoxylan with a main chain of 1,4-β-xylan and side chains of arabinose, (3) arabinan with the main chain and side chains of arabinose, and (4) β-1.3:1,4-glucan. The possible structures are shown in Figure 1. While Miura et al. [22] suggested further study to determine whether these were intermixing structures or parts of a composite molecule, there was no follow-up research.

Natural sugar analysis with chromatography was performed on various RBAC products by different authors [22,23,24,25], with the results summarised in Table 1. The subfraction II-6 of MeOH insoluble material of Biobran MGN-3 mentioned earlier has glucose (30.2%), galactose (23%), arabinose (22.2%), and xylose (13.7%) as the main components. As arabinoxylan comprises arabinose and xylose, the active ingredient of Biobran MGN-3 thus consists of complex polysaccharides with arabinoxylan as its primary structure (36%) while also containing galactan and glucan. However, the composition can vary drastically across different subfractions of Biobran MGN-3, with fraction IV in Table 1 consisting mainly of glucose (89.2%) [22].

RBEP (sample 1) from Erom was reported to have a much higher content of mannose (22.9%), similar in arabinose content (21.8%) but lower in xylose, glucose, and galactose contents compared to Biobran MGN-3 (Fr-II) [25]. However, in a separate study by Choi et al. [26], the primary sugar composition of RBEP was reported as xylose (22.25%) and glucose (11.71%), with only traces of galactose and arabinose detected (sample 2). Hence, the results from these two analyses were markedly different.

Furthermore, the two RBAC products from STR Biotech, namely BPP (a black rice bran derivative) and BPRBE, also appeared to have drastically different sugar compositions than Biobran MGN-3 and RBEP, as shown in Table 1 [23,24]. It is unclear how different sugar compositions may affect the immunomodulating capacity of the products in the absence of any direct comparison. Regardless, these RBAC products (Biobran MGN-3, RBEP, BPP, BPRBE) were reported to have enhanced macrophage-stimulation activities, with details presented in a later section.

### 3.2. Antioxidant Capacity

Reactive oxygen species (ROS), such as superoxide radicals (O_2_^•−^), hydrogen peroxide (H_2_O_2_), and hydroxyl radicals (•OH), are metabolic by-products of biological processes [27]. Low levels of ROS can be beneficial to the immune system. Phagocytes, such as neutrophils and macrophages, synthesise and release ROS in respiratory bursts to facilitate phagocytosis and the killing of microbial [28]. Excess production of ROS, however, can lead to oxidative stress that harm the host cells, damaging the mitochondria and deoxyribonucleic acid (DNA), thus inducing premature cell death or leading to neoplasm [29]. Therefore, it is widely accepted that oxidative damage caused by ROS is the primary cause of aging, although the theory is yet to be substantiated by research evidence [30]. An imbalance in ROS also plays a role in the dysfunction of immune cell activity, such as the adaptive immune responses by T and B cells [31,32], and excess ROS has been identified as a contributing factor to autoimmune disorders [33,34]. Therefore, maintaining ROS homeostasis is crucial for the immune system, health, and aging.

Antioxidants are stable molecules readily donating electrons to neutralise free radicals and hence counteract the effects of ROS [35]. The endogenous antioxidant system, which consists of enzymatic, hydrophilic, and lipophilic antioxidants, eliminates excess ROS to protect the host against oxidative stress, thus maintaining homeostasis [36]. The antioxidant activities of plant-based polysaccharides are known to improve markedly after chemical modification [37]. Specifically, fermentation and treatment with enzymes enhance the antioxidant capacity in rice bran by removing bonds between bioactive and cell wall components [38]. Therefore, RBAC, as a form of denatured hemicellulose of rice bran, may be a rich source of antioxidants needed for ROS balance. Table 2 summarises the research on the antioxidant capacity of RBAC.

Tazawa et al. [39] studied the free radical scavenging activities of Biobran MGN-3 using the compound and three of its fractions (L, M, S) extracted with fractional ethanol condensation. The study found high scavenging activities in Biobran MGN-3 and all its fractions. The scavenging rates were dose-dependent against O_2_^•−^ and •OH at 20, 2.0, and 0.2 mg/mL of the test samples. In particular, the S fraction of Biobran MGN-3 (<3000 molecules) was the most effective in the inhibition of •OH caused by O_2_^•−^ and ultraviolet irradiation [39].

In another study, An [40] evaluated the antioxidant capacity of RBEP compared to broccoli, a known high-antioxidant food, with two assays: the Oxygen Radical Antioxidant Capacity (ORAC, Cell Biolabs Inc., San Diego, CA, USA) and Total Antioxidant Status (TAS, Randox Labs. Ltd., Gortnagallon, Crumlin, UK). The study found the fat-soluble (lipophilic) sample of RBEP to have a higher antioxidant capacity than its water-soluble (hydrophilic) counterpart (446 vs. 326 μM Trolox equivalent/g). Both fat- and water-soluble RBEP had much higher ORAC values than lipophilic and hydrophilic extracts from raw broccoli, broccoli seed, and sprout. However, in the TAS assay, broccoli sprout extract had the highest measurement at 0.56 mmol/100 g, whereas RBEP ranked second with 0.4 mmol/100 g compared to 0.18 and 0.38 mmol/100 g of raw broccoli and broccoli seeds, respectively (An, 2011). Moreover, co-culturing L929 (mouse fibroblast) cell line with 1, 10, 100, and 500 μg/μL of RBEP for 72 h did not affect cell count. Hence, the study confirmed that RBEP was not cytotoxic and could be a functional food comparable to or even better than broccoli in antioxidant content.

Noaman et al. [41] investigated the in vivo antioxidant effect of RBAC. Adult female Swiss albino mice (*n* = 10) were injected intraperitoneally (i.p.) with 25 mg/kg body weight (BW) of Biobran MGN-3 six times a week over 25 days. These Biobran MGN-3-treated mice were found to have higher glutathione, an antioxidant, in the liver compared to untreated mice (*n* = 10) by 24.79% (*p* < 0.05) at the end of the study, even though the plasma glutathione level remained unchanged from the control values. Correspondingly Biobran MGN-3 appeared to significantly increase the production of glutathione peroxidase in the liver by 29.34% (*p* < 0.05) over control but not in the blood. Other endogenous antioxidant enzymes in the liver and plasma, including glutathione-S-transferase, superoxide dismutase and catalase, were no different from the control values. The study also found significant increases (*p* < 0.05) in the gene expression of glutathione peroxidase and catalase in Biobran MGN-3-treated mice compared to the control mice. So, Biobran MGN-3 is a potent antioxidant and could affect the production of endogenous antioxidants epigenetically, particularly glutathione and glutathione peroxidase to improve the host defence against ROS.

### 3.3. Absorption and Effects on Peyer’s Patches

The hemicelluloses of defatted rice bran are dietary fibres that resist digestion in the human intestines without prior processing [42]. The bioconversion using *L. edodes* mycelial enzyme breaks down the stable cross-link networks of polysaccharides making RBAC 98.4% water-soluble [22]. However, whether the resultant compounds can be absorbed into human intestines to exert biological reactions remains questionable.

Endo and Kanbayashi [43] addressed the bioavailability of RBAC with an in vivo experiment using healthy BALB/c mice. The mice were first injected (i.p.) with Biobran MGN-3 solution (1 mg/mL) emulsified with the same volume of complete Freund’s adjuvant. A subsequent 0.1 mL Biobran MGN-3 emulsified with the same volume of incomplete Freund’s adjuvant was also given to raise immunisation. The serum polyclonal antibodies against immunoreactive substances of Biobran MGN-3 were measured by enzyme-linked immunosorbent assay (ELISA). After this, the mice were fed 10 mg/kg BW of Biobran MGN-3 orally, and blood samples were collected at 0, 1, 2, and 3 h after oral administration. The study found significantly lower levels of polyclonal antibodies at 2 and 3 h after oral intake (Wilcoxon, *p* < 0.01), indicating the absorption of Biobran MGN-3 into the bloodstream through the digestive tract causing the polyclonal antibodies to bind to the polysaccharide antigens in Biobran MGN-3 [43]. Hence, some portion of Biobran MGN-3 is present within the bloodstream after digestion, triggering an antibody reaction in the host, demonstrating that Biobran MGN-3 is a source of pathogenic-associated molecular pattern (PAMP) molecules that could elicit immune responses [44]. Notwithstanding, it remains unclear how much Biobran MGN-3 can be absorbed, the structures that trigger the immune reaction, and whether these structures are responsible for its biological effects.

**Figure 2:**
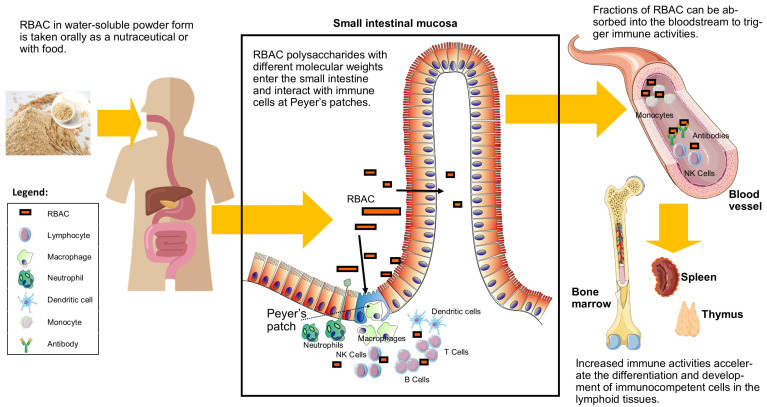
A graphical illustration of RBAC absorption in the small intestines after oral consumption. The compound interacts with the immune cells at the Peyer’s patches, with some fractions entering the bloodstream to exert further immunological effects.

Regardless of its absorption rate, RBAC could also stimulate the intestinal immune system via Peyer’s patches when transiting through the lowest portion of the small intestine. The in vitro effects of RBEP on Peyer’s patch were investigated by Kim et al. [45]. Peyer’s patch cells were isolated from the small intestine of C3H/He mice and cultured with the bone marrow cells for six days. The growth capacities of the bone marrow cells were evaluated when incubated with RBEP, bacteria lipopolysaccharide (LPS, an endotoxin as a positive control), and saline (control). As a result, RBEP stimulated bone marrow cell growth by 109% compared to the control and at a level similar to LPS. Therefore, the digestion of RBAC could potentially accelerate the haematopoietic responses of the bone marrow cells to differentiate into immunocompetent cells via activating Peyer’s patches. Figure 2 illustrates RBAC’s interactions with the immune cells in the small intestine before and after absorption.

### 3.4. Macrophage Stimulation and Phagocytosis

Macrophages are derived from the circulating monocytes originating from the bone marrow. As a type of professional phagocytic cell, macrophages embed in tissues and engulf, digest, and eliminate invading microorganisms, foreign bodies, and damaged or mutated cells while initiating proinflammatory responses if necessary [46,47]. Hence, macrophages are an essential component of the innate defence mechanism of the host tissues. Additionally, macrophages play a role in antigen presentation, triggering adaptive immune responses from the cluster differentiation (CD) 8+ T lymphocytes during infection and inflammation [48]. After pathogen elimination, macrophages actively assist in wound healing by dampening inflammation, clearing of cell debris, and coordinating tissue repair [49]. Hence, there are two opposing phenotypes of macrophages: M1 and M2. M1 fights infections and may cause cellular destruction and tissue damage, whereas M2 activity fixes the damage and promotes tissue repair [50]. Plant-based polysaccharides are known to trigger immune responses in M1 macrophages with increased NO production for pathogen killing and cytokine secretion for inflammatory process regulation [51]. As such, the macrophage stimulation intensity is commonly used as an immunomodulating indicator of the strength of RBAC products or components [22,25]. Table 3 summarises the effects of RBAC on macrophages based on available in vitro and in vivo evidence.

Yu et al. [25] compared the macrophage stimulation activity of modified rice bran from the submerged culture of nine fungi (*Grifola frondose, L. edodes, Cordyceps militaris, Clonorchis sinensis, Agaricus blazei, Flammulina velutipes, Auricularia auricula-judae, Schizophyllum commune*, and *Coriolus versicolor*). Peritoneal macrophages (P-M∅) were isolated from male ICR mice and cultured with 10 and 100 μg/mL of rice bran modified by enzymes of different mushrooms. All modified rice bran showed higher macrophage stimulation activities measured with acid phosphatase (a lysosomal enzyme with a direct role in microbe killing and antigen presentation) when cultured at 100 μg/mL compared to saline control. Notwithstanding, the *L. edodes* product (RBEP) demonstrated the highest activity, followed by those produced with *G. frondose, S. commune*, and *C. versicolor*. Furthermore, RBEP’s macrophage stimulation level was similar to LPS at 10 μg/mL and higher at 100 μg/mL.

Biobran MGN-3 was shown to stimulate M1 macrophages to enhance the rate of attachment and phagocytosis of yeast (*Saccharomyces cerevisiae*), increase the production of proinflammatory cytokines (TNF-α and IL-6), and raise NO release in a dose-dependent manner (1 < 10 < 100 μg/mL). Ghoneum and Matsuura [54] demonstrated these effects in vitro by utilising three different macrophage cell types: human (U937), murine (RAW264.7), and P-M∅ freshly prepared from female C3H/HeN mice. Albeit, the responsiveness varied across cell lines. The macrophage stimulation activity of Biobran MGN-3 was also validated based on NO and cytokine production (TNF-α and IL-1β) by Miura et al. [22] while investigating the chemical structure of active ingredients of Biobran MGN-3.

Similar observations were made by Chae et al. [55] while comparing the immunomodulating effects of Biobran MGN-3 compared to polysaccharide-peptide (PSP) extracted from *C. versicolor*. The phagocytic activities of the P-M∅ extracted from Balb/c mice orally fed with 1.5 mg/L of either Biobran MGN-3 (69%) or PSP (65%) against *Candida parapsilosis* were significantly higher (*p* < 0.05) than P-M∅ of control mice fed with saline only (47%). Furthermore, both Biobran MGN-3 and PSP were observed to increase NO production in the RAW264.7 cell line, but Biobran MGN-3 appeared to be less effective than PSP (1.0 NO_2_-mM vs. >2.0 NO_2_-mM at 1000 μg/mL).

Regarding in vitro cytokine production in RAW264.7 macrophage cells, Biobran MGN-3 (1000 μg/mL) induced higher levels of IL-6 and TNF-α production than control but less than the levels influenced by PSP and LPS and was found to increase the major histocompatibility complex (MHC) class II expression in RAW264.7 cells compared to control after two days of incubation but also at a level lower than PSP, LPS, and interferon (IFN)-γ [55].

Biobran MGN-3 also upregulated human phagocytic cells, including monocytes, the precursors of macrophages, and neutrophils, to enhance intracellular killings of microbes, as Ghoneum et al. [56] reported. Treatment with Biobran MGN-3 (100 μg/mL) significantly improved (*p* < 0.01) the phagocytosis of *Escherichia coli* by monocytes (110%) and neutrophils (400%) in human peripheral blood lymphocytes (PBL) compared to control. Increased oxidative burst by monocytes and neutrophils with elevated H_2_O_2_ production in the presence of *E. coli* was observed. Culturing human macrophage cells (U937) with Biobran MGN-3 (1, 10, 100, 1000 μg/mL) also dose-dependently increased TNF-α, IL-6, IL-8, and IL-10 production with significant differences (*p* < 0.01) observed from 10 μg/mL onward, compared to control. However, Biobran MGN-3 was not bactericidal as no in vitro activity was observed against 31 strains of selected anaerobic and microaerobic bacteria [56].

The stimulating effect of RBEP on macrophages was also confirmed by Kim et al. [45] in vitro and in vivo by measuring the lysosomal enzyme level. P-M∅ were extracted from male ICR mice and cultured with 10 and 100 μg/mL of RBEP. Both doses elicited more than twice the control activity level, similar to the levels of LPS under the same dose. The effects were also replicated in vivo with C3H/HeN mice fed with RBEP (50 and 250 mg/kg BW) for 5 days showing a 1.41- and 1.44-fold increase in P-M∅ lysosomal enzyme activity compared to the control.

Another study also reported that EFR (Erom’s fermented rice bran) possessed macrophage stimulation activity through experiments with P-M∅ (C57/BL6 mice) and RAW264.7 cell line. Dose-dependent significant increases (*p* < 0.05) in NO production were observed by Kang et al. [53] when RAW264.7 cells were treated with 0.5, 1, 2, 4, and 8 μg/mL of EFR, compared to untreated control. The level of NO production in 8 μg/mL of EFR was comparable to that of 1 μg/mL of LPS. At the gene expression level, the mRNA expression of inducible nitric oxide synthase (iNOS), a key marker of M1 macrophage activation (Xue et al., 2018), was detected by reverse transcriptase (RT) polymerase chain reaction (PCR) to increase in a similar trend under different EFR treatment concentrations. RAW264.7 cells treated with EFR (0.5–8 μg/mL) also demonstrated a significant increase (*p* < 0.05) in IL-1β, IL-10, IL-6, and TNF-α secretion dose-dependently when compared to control. The lysosomal enzyme activity of P-M∅ treated with EFR was significantly increased (*p* < 0.05) to 134.9–142.2% at a concentration of 0.5–8 μg/mL relative to the control. Such levels are at par with the 135.8% increase demonstrated by LPS [53].

Kim et al. [52] investigated the macrophage stimulation abilities of three variants of RBAC from STR Biotech: CFP (a crude fermentation-polysaccharide CFP extracted from black rice bran cultured with *L. edodes*), a secondary CFP product fermented with *Lactobacillus gasseri* (CFP-L), and another secondary CFP extract from fermentation with baker’s yeast, *S. cerevisiae* (CFP-S). P-M∅ extracted from Balb/c mice orally administered with 250 mg/kg BW/day of treatment for 4 weeks showed lysosomal enzyme activities of 104.60 ± 10.97% (CFP), 115.21 ± 18.94% (CFP-S), 97.99 ± 16.79% (CFP-L) relative to control. In the presence of LPS, however, macrophage activity significantly increased (*p* < 0.05) for CFP (113.21 ± 13.10%) and CFP-S (138.78 ± 24.41%) but decreased for CFP-L (93.60 ± 15.34%). Hence, the macrophage stimulation effect of CFP was enhanced by a secondary fermentation with *S. cerevisiae* but dampened by adding *L. gasseri*.

Kim et al. [24] found that polysaccharides extracted from a combined culture of black rice bran and *L. edodes* (BPP from STR Biotech) could increase the lysosomal enzyme activity level of P-M∅ (BALB/c mice) in vitro by about 5.4-fold at 10 μg/mL (15% to 81%) and 2.5-fold (49% to 120%) at 100 μg/mL, compared to the polysaccharides of *L. edodes* without black rice bran. The macrophage stimulation activity of BPP at 100 μg/mL was also higher than the level stimulated by 100 μg/mL of LPS. Although BPP was not bactericidal in vitro against *Salmonella typhimurium*, RAW 264.7 macrophages pretreated with 1, 10, and 100 μg/mL BPP demonstrated 1.4-, 2.4-, and 3.5-fold increases in bacterial uptake rates against *S. typhimurium* compared to untreated macrophages, respectively (Kim et al., 2014). The phagocytotic stimulatory effect of BPP was also tested in vivo. In female BALB/c mice inoculated with *S. typhimurium* in the peritoneal cavity, pretreatment with 10 mg/kg BW of BPP orally or i.p. showed significantly reduced (*p* < 0.05) bacteria growth compared to control.

Further experiments also revealed that BPP, at 100 μg/mL, could induce RAW 264.7 monocyte/macrophage cells to morphological change into the dendrite-like structure, reaching up to 3.6-, 10.3-, and 12.4-fold changes after 2, 4, and 8 h of incubation (Kim et al., 2014). It was observed that BPP-treated cells (RAW 264.7) that were subsequently infected with *S. typhimurium* showed more significant intracellular bacteria presence (*p* < 0.05) after 2 h compared to the control. In contrast, the bacterial count markedly decreased (*p* < 0.05) relative to the control after 4 and 8 h. The results suggested an initially enhanced engulfment of bacteria by the BPP and a subsequent switch to intracellular bacteriolysis through activation of T helper type 1 (Th1) cell cytotoxic reaction initiated and directed by M1 macrophages over time [57,59].

BPRBE from STR Biotech was also shown to significantly increase (*p* < 0.05) NO production of RAW 264.7 macrophage cells compared to non-bioprocessed rice bran extract and vehicle control [23]. The NO formation levels were dose-dependent (1, 10, and 100 μg/mL), and at 10 μg/mL or greater doses, they were significantly higher than that obtained with LPS [23]. Through RT-PCR and western blot analysis, Kim et al. [23] confirmed mRNA expression level of the iNOS gene of *Salmonella*-infected RAW 264.7 cells pretreated with BPRBE was about 1.9-fold higher than that in the infected control cells without treatment. The phagocytotic effects of BPRBE on *S. typhimurium*-infected RAW 264.7 cells were also enhanced significantly (*p* < 0.05) with 1.3-, 2.3-, and 3.4-fold rate increment compared to control with 1, 10 and 100 μg/mL BPRBE treatment, respectively.

**Figure 3:**
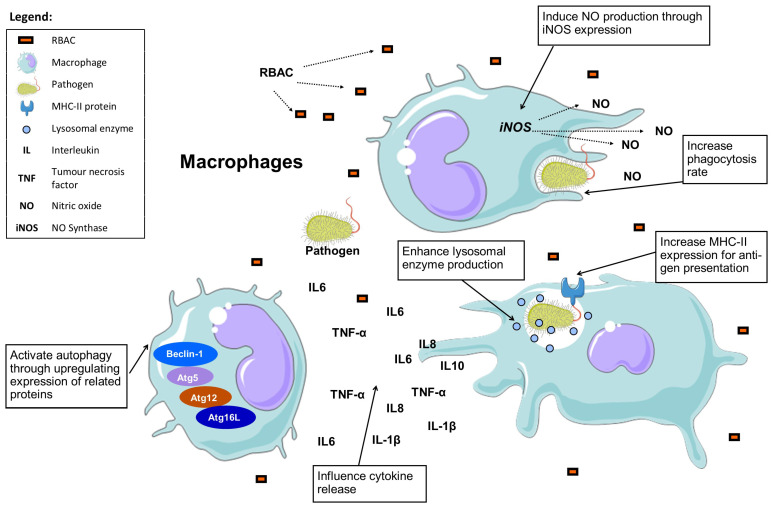
RBAC could enhance macrophages’ lysosomal enzyme and nitric oxide production while increasing their phagocytosis rate against pathogens. RBAC also further influences cytokine release and cross-presenting antigens to activate follow-up immune responses while triggering autophagy of infected cells (Boxed items are the effects of RBAC).

*Salmonella* was known to downregulate the expression of the autophagy-related membrane proteins (Beclin-1, Atg5, Atg12, Atg16L) of infected cells to evade autophagic recognition. Through, Western blot analysis, BPRBE treatment was found to upregulate the expression of these autophagy-related proteins regardless of bacterial infection [23]. Additionally, BPRBE treatment (100 μg/mL) also markedly induced (*p* < 0.05) the production of IFN-β in the *Salmonella*-infected macrophage cells via the interferon regulatory transcription factor 3 (IFR3) pathway [23]. In short, RBAC possesses remarkable macrophage-stimulating effects based on the available in vivo and in vitro evidence, as depicted in Figure 3.

### 3.5. Natural Killer Cell Activity

Natural killer cells (NKC), representing about 5–20% of circulating lymphocytes in humans, are large granular lymphocytes developed and matured in the bone marrow and secondary lymphoid tissues such as tonsils, spleen, and lymph nodes [60]. As part of the innate immune system, NKC are naturally cytotoxic and can detect and destroy viral-infected or tumorigenic cells without priming by expressing a myriad of activating and inhibitory cell surface receptors [61]. Hence, harnessing NKC’s killing power has been a strong interest in suppressing malignant neoplasm since its first discovery in the 1960s [62]. The bioactivities of RBAC on NKC have been studied extensively in vitro and in vivo. Table 4 summarises the effects of RBAC on NKC activity based on the available preclinical evidence.

In vitro experiments with the PBL from healthy donors (*n* = 6) cultured with Biobran MGN-3 showed significant increases (*p* < 0.001) in NKC cytotoxicity tested with chromium (^51^Cr) release assay. The effect was dose-dependent (130% at 25 μg/mL vs. 150% at 100 μg/mL) [63]. Biobran MGN-3 also increased IFN-γ production significantly (*p* < 0.001), showing 340, 390, and 580 pg/mL of IFN-γ production at 25, 50, and 100 μg/mL, respectively, compared to <100 pg/mL in control. Quantification of total NKC, however, indicated no increase in the percentage of NKC populations in PBL cultured with Biobran MGN-3 of all concentrations as identified by the CD56+/CD3− and CD16+ markers [63]. Hence, Biobran MGN-3 increased the cytotoxicity of existing NKC but not the cell count.

A follow-up study on the in vitro effects of Biobran MGN-3 on NKC by Ghoneum and Jewett [64] found significant increases in NKC cytotoxicity in both PBL (*p* < 0.001) and purified NKC (*p* < 0.01) by Biobran MGN-3 (500 μg/mL). The NKC augmentation effect of Biobran MGN-3 was enhanced by co-culturing with IL-2 (500 U/mL) in PBL but not in purified NKC. Biobran MGN-3 (100 and 1000 μg/mL) was also shown to significantly increase (*p* < 0.001) the secretion of TNF-α in PBL samples collected from 25 donors in a dose-dependent manner. However, there were variations across individuals with responses categorised into three groups at 1000 μg/mL: ≤20-fold (40%, *n* = 10), 20 to 100-fold (24%, *n* = 6), and >100-fold (36%, *n* = 9). TNF-α secretion by PBL cultured with Biobran MGN-3 (1000 μg/mL) was also significantly enhanced (*p* < 0.001) with the presence of IL-2. In contrast, TNF-α secretion in purified NKC treated with Biobran MGN-3 (1000 μg/mL) did not increase further with the addition of IL-2. These experiments showed that while Biobran MGN-3 worked synergistically with IL-2 to increase NKC cytotoxicity and TNF-α in PBL, Biobran MGN-3 alone could enhance the NKC cytotoxicity, as demonstrated by its effects on purified NKC [64].

Production of IFN-γ by PBL was tested with samples from 14 donors by Ghoneum and Jewett [64]. Again, Biobran MGN-3 (100 and 1000 μg/mL) treatment significantly increased (*p* < 0.03) IFN-γ secretion with variation across individuals. Adding IL-2 resulted in a synergistic increase (*p* < 0.03) in IFN-γ production in all samples, and the effect was dose-dependent. Furthermore, a synergistic increase in IFN-γ production (*p* < 0.01) was also observed when adding IL-2 to Biobran MGN-3 treatment of purified NKC.

Ghoneum and Jewett [64] further explored the mechanisms of NKC activation by Biobran MGN-3. CD69 cell surface receptor, CD54 (intercellular adhesion molecule-1, ICAM-1), and CD25 (IL-2 receptor alpha chain) expressions of NKC were all observed to be upregulated in PBL treated with Biobran MGN-3 comparable to those with IL-2. Upregulation of CD69, CD54, and CD25 was also observed in other lymphocytes after Biobran MGN-3 treatment but to a lower extent.

The enhancement of depleted NKC activity in aged C57BL/6 and C3H female mice (18 months old) by Biobran MGN-3 was explored by Ghoneum and Abedi [65]. Biobran MGN-3 (0.1 mL with 10 mg/mL, i.p.) was reported to significantly increase the cellularity (404–470%, *p* < 0.01) and NKC activity against YAC-1 target cells (4.8–6-fold increase, *p* < 0.01) in the peritoneal cavity after 2 days with the levels remained elevated through to the 14th day. However, the treatment did not increase NKC activity in the spleen or the bone marrow, even though a significant increase in splenic cellularity was observed (145–192%, *p* < 0.025). In contrast, oral feeding of Biobran MGN-3 at the same dose elicited a 200% increase (*p* < 0.01) in splenic NKC activity in C57BL/6 mice 14 days after treatment but did not affect peritoneal NKC activity and cell count compared to the control. For comparison, the in vitro NKC activity of splenic lymphocytes isolated from aged C57BL/6 mice exhibited a 4-fold increase (*p* < 0.01) over the control after culturing with Biobran MGN-3 at 25 and 100 μg/mL.

Furthermore, the peritoneal NKC of C57BL/6 mice (*n* = 3) demonstrated a remarkable increase in BLT-esterase activity (*p* < 0.01) and granularity after 5 days of i.p. treatment, which were alternative markers for increased cytotoxicity [65]. The binding capacity of peritoneal NKC of mice treated with Biobran MGN-3 was also reported to have significantly higher percentage conjugates (26% vs. 13%, *p* < 0.01), a 2-fold increase, compared to the control. Ghoneum and Abedi [65] also found the presence of P-M∅ could substantially lower (*p* < 0.025) the in vivo peritoneal NKC activity.

Badr El-Din et al. [66] investigated the in vivo effect of Biobran MGN-3 on NKC activity in young female Swiss albino mice (2 months old) administered with 100 μg/mL/day of Biobran MGN-3 with intramuscular injection for 14 days. The splenic NKC activity was measured against the YAC-1 tumour target cells. The results showed a significant increase in NKC activity in the treated mice (27.1 [lytic units] LUs vs. 8.3 LUs, *p* < 0.01) compared to the control. Furthermore, a two-fold increase (27.5% vs. 14%, *p* < 0.01) in the NKC binding capacity in terms of the percentage of conjugate formation to target cells was also detected.

Likewise, the splenic NKC activity (against YAC-1 target cells) of male Lewis rats fed with 250 mg/day of Biobran MGN-3 as a dietary supplement after two weeks was also enhanced significantly (*p* < 0.045) compared to rats fed with only a control diet, as reported by Giese et al. [67]. Such an increase in activity was associated with elevations of IL-2 (*p* < 0.05) and IFN-γ (*p* < 0.08) production in splenocytes. The effect of RBEP on the splenic NKC activity of healthy mice was investigated by Kim et al. (2007). Male ICR mice (4 weeks old) were fed orally with 50 mg/kg BW or 250 mg/kg BW of RBEP for 5 days. RBEP was shown to enhance the NKC activity against YAC-1 cells significantly, reporting 32.8 LUs (50 mg/kg, *p* < 0.05) and 46.3 LUs (250 mg/kg, *p* < 0.01) compared to the 22.8 LUs of the controls.

**Figure 4:**
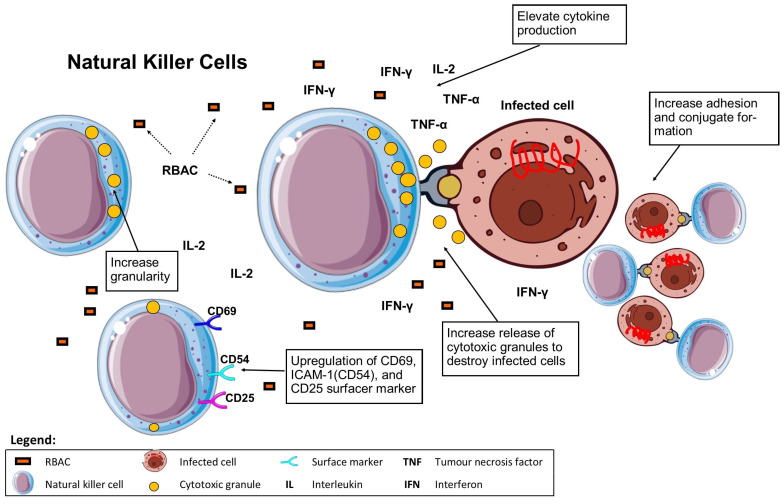
RBAC upregulates NKC cytotoxicity with increased granularity, costimulatory marker (CD69, ICAM-1, and CD25) expression, cytokine production, conjugate formation, and release of cytotoxic granules to destroy infected cells (Boxed items are the effects of RBAC).

Pérez-Martínez et al. [58] conducted in vitro cytotoxicity assays of NKC isolated from PBL of healthy volunteers against multiple cancer cell lines (K562, NB1691, Jurkat, A673, A-04, RD, RH30). Significant increases in NKC cytotoxicity against all tested cell lines (*p* < 0.05) compared to resting NKC were reported after overnight stimulation with Biobran MGN-3 (100 μg/mL). The cytotoxicity measured in percentage lysis of treated NKC ranged from 34% to 80% in different cell lines compared to 13% to 69% of the resting NKC. Such levels were less than IL-5 (10 ng/mL) stimulated NKC. Nonetheless, Biobran MGN-3 (100 μg/mL) was found to have a synergistic effect in significantly enhancing the stimulatory effect (*p* < 0.05) of low dose IL-2 (40 IU/ml) on NKC cytotoxicity level to that obtained with 1000 IU/mL of IL-2. Regarding phenotyping, NKC stimulated with Biobran MGN-3 showed increased expression of CD69 and CD25 by 3.1-fold and 3.2-fold over resting NKC (measured in mean fluorescence intensity), respectively. However, Biobran MGN-3 did not affect the expression of other receptors on NKC, including NKG2D (natural killer group 2D), DNAM (DNAX accessory molecule), NCRs (nuclear receptor coregulators) and TLRs (toll-like receptors), unlike IL-5, the positive control [58]. The biological effects of RBAC on NKC are summarised in Figure 4.

RBAC’s ability to enhance NKC activity in a human clinical trial was first reported by Ghoneum [63]. Healthy adults (*n* = 24) were divided into three groups and received Biobran MGN-3 in doses of 15, 30, and 45 mg/kg BW/day (*n* = 8 in each group) for two months. The NKC activities in PBL were tested against K562 tumour cells at baseline and after 1 week, 1 month, 2 months, and 3 months. Biobran MGN-3 treatment at all concentrations demonstrated enhanced NKC cytotoxicity against K562 over time with significant differences over baseline achieved at 1 week (∼3.1-fold, *p* < 0.001) for 30 and 45 mg/kg dose and after 1 month (2-fold) for 15 mg/kg dose. Peak response was recorded after 2 months (∼5-fold) for all doses, and the NKC activity returned to baseline after 1 month of discontinuing treatment. Furthermore, after 1 month of treatment at 45 mg/kg BW/day, the binding capacity of NKC to K562 tumour targets, measuring in the percentage of conjugate formations, increased significantly over baseline (38.5% vs. 9.4%, *p* < 0.005). A similar trend of augmented NKC effect was also observed against the more resistant Raji tumour cells with 45 mg/kg dose [63].

Two other human studies also evaluated RBAC’s ability to enhance NKC activity in healthy adults. Ali et al. [69] recruited 20 adult volunteers (mean age = 33.6 ± 13.2) randomly assigned to take either 1 g/day or 3 g/day of Biobran MGN-3 for 60 days. Peripheral blood-derived mononuclear cells (PBMC) from these participants were obtained at baseline, 48 h, 1 week, 30 days and 60 days (5 time points) for evaluation. The results showed a significant effect for time (*p* = 0.001) based on repeated measure analysis of variance (ANOVA) on the NKC activity against K562 cell lysis but not for the group and the interaction of group and time. Total NKC activity peaked at 1 week and was significantly higher than the values at other time points. Unlike the previous study by Ghoneum [63], the NKC activity did not remain elevated throughout the treatment period but dropped back to baseline levels at 30 and 60 days.

Choi et al. [26] investigated the effects of RBEP on the NKC activity compared to placebo in healthy participants in a double-blind, randomised controlled trial (RCT). The participants in this study (*n* = 80) were randomly assigned to consume either 3 g/day of RBEP or placebo powder for 8 weeks. NKC activity against K562 cells was tested with the PBL of participants collected from baseline, 4 weeks, and 8 weeks. This study, however, did not find any significant effect of RBEP supplementation over placebo at all time points. Such a finding was consistent with what was reported by Ali et al. (2012) since any initial stimulating effects by RBAC would have subsided after 4 weeks, contrasting the findings of Ghoneum [63]. Possible explanations could be individual differences, with some healthy participants more receptive to the NKC stimulation effect of RBAC or product differences. Hence, more research on how RBAC could affect the NKC of healthy adults is warranted.

Biobran MGN-3 could also restore NKC activity in individuals with weakened immunity due to exogenous sources. Ghoneum [70] reported a study among 11 individuals who had workplace chemical exposure and received Biobran MGN-3 at 15 mg/kg BW/day for 4 months. The participants had low levels of NKC activity (10.2 ± 4.2 LUs) at baseline, but treatment with MGN-3 increased NKC activity 4- and 7-fold at 2 and 4 months, respectively.

Among older adults under institutional care (age 75–90), consuming Biobran MGN-3 (500 mg/day) for two weeks did not significantly affect the NKC compared to control according to a cross-over randomised RCT (*n* = 32) by Tazawa et al. [71]. However, a subgroup analysis by Maeda et al. [72] of the same study showed that those with low NKC activity at baseline (*n* = 12, ≤30%) had a higher increment in activity during Biobran MGN-3 treatment (34.1% increase) compared to the control (15.6% increase). However, the between-group difference was not statistically significant.

In another study with a younger cohort of older people, Elsaid et al. [73] showed that consuming Biobran MGN-3 could enhance NKC activity after 1 month in a placebo-controlled RCT. The study recruited 12 participants over 56 years old and randomly assigned to take either 500 mg/day of Biobran MGN-3 or a placebo (*n* = 6 in each group). Blood samples were analysed for NKC expressing CD107a, a functional marker for NKC activation, before and after the trial. The median CD107a expression in the Biobran MGN-3 significantly increased from 60.5% to 83.0% posttreatment (Wilcoxon, *p* < 0.046), whereas no significant differences were detected in the placebo group. However, the percentage of NKC in blood did not differ between groups and over time [73].

Such findings were also confirmed by Elsaid et al. [74] in a study with randomly selected geriatric volunteers (age ≥ 56) from a larger placebo-RCT (*n* = 80). Twelve participants who took 500 mg/day of Biobran MGN-3 or placebo (6 in each group) were tested for NKC CD107a expression before treatment and after study completion at 3 months. Mean NKC CD107a expression significantly increased (*p* = 0.004) from 49.5 ± 10.4% to 75.2 ± 6.6% for the Biobran MGN-3 group compared to insignificant difference pre- and posttreatment values in the placebo group (45.3 ± 12 vs. 50.8 ± 19.5). Hence, the enhanced NKC activity stimulated by RBAC could be sustained for 3 months in the geriatric group. Table 5 summarises RBAC’s effects on the NKC activity of healthy adults with evidence from human clinical trials.

### 3.6. Dendritic Cell Maturation

Dendritic cells (DC) are considered professional antigen-presenting cells in the immune system, alongside macrophages and B cells [75]. Like macrophages, DC sense and engulf invading pathogens [76]. Instead of directly destroying an invading pathogen, DC communicate to T lymphocytes by antigen presentation to MHC complexes or other cell surface molecules, initiating long-lasting antigen-specific responses [77]. Furthermore, DC also modify ongoing immune responses through the secretion of cytokines and growth factors while interacting with other immune cells, such as NKC [78]. As such, DC are regarded as master regulators of the immune system, which play a critical role in bridging innate to adaptive immune responses [77]. DC can be found in practically all tissues but remain in the ‘immature’ state until tissue homeostasis disturbances are triggered by PAMP, damage-associated molecular patterns, proinflammatory cytokines, or pathogens. Maturation involves significant changes in surface proteins, intracellular pathways and metabolic activity, together with the migration of DC from peripheral tissue to secondary lymphoid organs where T lymphocyte activation may occur [78].

RBAC also possesses the capacity to upregulate the maturation of DC with evidence from research conducted on monocyte-derived DC, as summarised in Table 6.

Cholujova et al. [79] investigated the modulatory effects of RBAC on human DC differentiation and maturation using monocyte-derived DC in vitro. Immature DC (iDC) were derived from peripheral monocytes isolated from the buffy coat blood of healthy human donors. Cytokine maturation mix (CMM1) (TNF-α, IL-1β and IL-6) or CMM2 (LPS and IFN-γ) was used to induce DC maturation co-cultured with different concentrations of BioBran MGN3 (0, 10, 100, 400 and 1000 μg/mL) to assess the maturation processes. The study found BioBran MGN3 down-regulated the expression of CD14 (a monocyte marker) and CD1a (an antigen-presenting molecule) on the surface of iDC while markedly increasing CD83 expression (a DC maturation marker) in a dose-dependent manner.

Similar observations were made in both matured DC samples (matDC1 and 2, matured with CMM1 and 2, respectively) with higher intensity of up- or down-regulation compared to the level seen in iDC using the same dose of BioBran MGN3 [79]. Furthermore, the endocytic activity of iDC (evaluated by the uptake of FITC-conjugated dextran at 37 °C) also reduced from 73% to 27.7% (100 μg/mL), 17.7% (400 μg/mL), and 14.4% (1000 μg/mL) under different concentrations of BioBran MGN3, approaching the levels of mDC (14.9% for matDC1 and 5.6% for matDC2). BioBran MGN3 increased the surface density of costimulatory molecules CD80 and CD86 on iDC and further enhanced the CD80 and CD86 density on matDC1 and matDC2. Furthermore, the study also found iDC, matDC1, and matDC2 expressed higher levels of CD123 (IL-3 receptor α chain) and lower levels of CD11c cell surface antigens, the phenotype represented by CD123+/CD11c- plasmacytoid DC, in the presence of BioBran MGN3 [79].

In parallel, the stimulatory effects of BioBran MGN3 were also validated by Ghoneum and Agrawal [80] utilising the monocyte-derived DC isolated from PBMC of healthy donors. DC were treated for 24 h with BioBran MGN3 (0, 5, 10 and 20 μg/mL) or LPS (1 μg/mL) as a positive control. Activation of DC was determined by assessing the expression of costimulatory and maturation markers (CD40, CD80, CD83, CD86 and HLA-DR). Flow cytometry analysis showed a dose-dependent upregulation in CD83 and CD86 surface markers similar to the effects of LPS on DC. Moreover, the BioBran MGN3 treated DC also produced significantly higher levels (*p* < 0.05) of IL-1β, IL-6, IL-10, TNF-α, IL-12p40, IL-12p70, and IL-2, compared to untreated DC. In comparison, LPS also significantly induced the production of these cytokines in DC, except IL-1β. In particular, BioBran MGN3 increased the production of IL-1β from DC in vitro in a dose-dependent manner [80].

The BioBran MGN3-activated DC also demonstrated the capacity to boost T-cell proliferation in vitro [80]. Co-culturing of DC pretreated with 10 μg/mL BioBran MGN3 with allogeneic CD4+ T cells increased the proliferation by 1.4-fold, a 73.6% increase compared to CD4+ T cells cultured with untreated DC. Such activation level is similar to that of DC activated with LPS. Furthermore, the study also detected a 1.3-fold increase in the expression of CD25 markers on the T lymphocytes co-cultured with BioBran MGN3 (10 μg/mL) induced DC compared to the control. In terms of cytokine production from T lymphocytes, significant increases (*p* < 0.05) in IFN-γ, IL-10, and IL-17 were reported in Biobran MGN-3 (10 μg/mL) primed DC-induced T lymphocytes compared to control [80].

**Figure 5:**
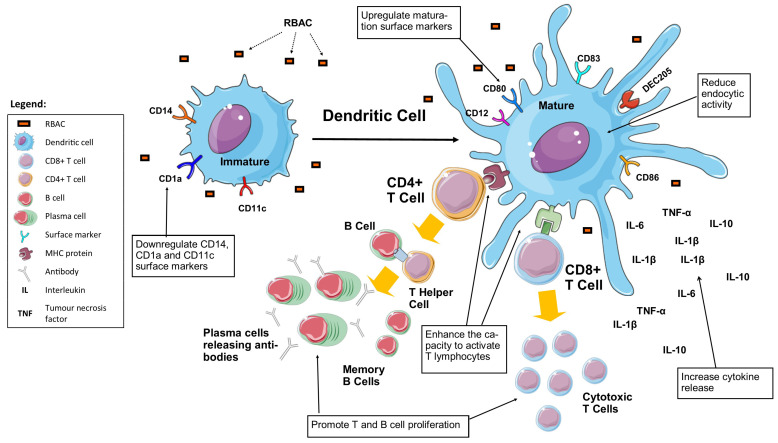
RBAC stimulates the maturation of dendritic cells by downregulating the immature surface markers, reducing endocytic activities while upregulating the maturation markers. RBAC-stimulated dendritic cells have increased cytokine production and an enhanced capacity to activate CD4+ and CD8+ lymphocytes leading to the proliferation of T and B cells for adaptive immune responses (Boxed items are the effects of RBAC).

In a follow-up study, Ghoneum and Agrawal [81] continued to explore the effects of BioBran MGN3-primed DC on cytotoxic CD8+ T lymphocytes. Again, monocyte-derived DC isolated from PBMC of healthy donors were incubated with BioBran MGN3 (20 and 40 μg/mL) for 24 h. DC treated with BioBran MGN3 showed signs of maturation with increased DEC-205 expression for antigen-presenting in a dose-dependent manner. Treatment of 20 μg/mL of BioBran MGN3 also significantly increased IL-29 production in DC (*p* < 0.05) compared to the control. At the same time, increased secretion of IFN-α and IFN-β were also detected but did not achieve statistical significance. Culturing the activated DC (by 20 μg/mL BioBran MGN3) with purified, allogeneic CD8+T cells for 7 days resulted in significantly higher levels of granzyme B-positive CD8+ T cells (*p* < 0.05) as compared to unstimulated DC treated CD8+ T cells. Granzyme-expressing CD8+ T cells possess an increased capacity to kill tumour cells. Experiments by Ghoneum and Agrawal [81] further demonstrated that CD8+ T cells cultured with BioBran MGN3-activated DC had increased cytotoxicity against tumour cell targets significantly (*p* < 0.05) compared to those treated with unstimulated DC. Figure 5 is a graphical illustration of the maturation of DC under the influence of RBAC.

### 3.7. T and B Cell Proliferation

The T and B cells are the two main types of lymphocytes of the adaptive immune response, which are antigen-specific. A distinct T and B cell population recognises and responds to a specific antigenic epitope [82]. Developed from stem cells in bone marrow while matured in the thymus, naïve T cells constantly migrate to secondary lymphoid organs, including lymph nodes, tonsils, spleen, and Peyer’s patches, where they can encounter antigen presented by the antigen-presenting cells such as DC and macrophages. Once activated, naïve T cells differentiate into effector cells with specialised phenotypes. Among them are cytotoxic CD8+ T cells, which can attack and destroy malignant or virus-infected cells via apoptosis induction, and CD4+ T cells are T helper cells that produce cytokines and stimulate B cells to differentiate into antibody-secreting plasma cells [82]. B lymphocytes are also developed and differentiated from stem cells in bone marrow, but unlike their T counterpart, B cells continue to mature in the bone marrow. Mature B cells also migrate to secondary lymphoid organs and can transform into plasmocytes for producing specific antibodies after activation [82].

As presented in the previous section, RBAC is an inducer of DC maturation to initiate the adaptive immune response of T and B cells. Ghoneum [70] also observed in participants (*n* = 11) with chemical exposure the increase of T and B cell mitogens by 130–150% compared to baseline after consuming Biobran MGN-3 (15 mg/kg/day) for 4 months (Table 5). Several preclinical studies confirmed RBAC’s effects on enhancing T and B cell proliferation in the secondary lymphoid organs. A summary of the evidence on RBAC’s capacity to promote splenic T and B cell proliferation is shown in Table 7.

Using rosette-forming cell assay, Bae et al. [83] reported that the splenocytes of BALB/c mice fed with 1.5 mg/day of Biobran MGN-3 for 10 days could significantly induce rosette formation due to higher antibody production by 30% (*p* < 0.005) compared to the control and the level was also higher than splenocytes of mice fed with PSP (1.5 mg/day). Similarly, the splenocyte plague formation was also highest in the Biobran MGN-3 group, with a 14% increase over the control group, compared to 11% higher than the control in the PSP group. Hence, these results showcase the in vivo capacity of Biobran MGN-3 in B cell activation and T cell proliferation [55,83].

Biobran MGN-3 also exhibited in vitro effects in inducing splenocyte proliferation in a dose-dependent manner (1, 10, 100, 1000 μg/mL), albeit at a much lower level than LPS (which proliferated B cells specifically), concanavalin A (Con A, a T cell proliferation inducer), and PSP. Biobran MGN-3 also induced IFN-γ secretion like LPS in splenocytes [83]. Giese et al. [67] confirmed such observations in vivo with male Lewis rats fed with Biobran MGN3 (0.25 g/day) for 14 days. Splenocytes of Biobran MGN3-fed rats (*n* = 12) showed significantly higher proliferative activity against the superantigen toxic shock syndrome toxin-1 compared to those from the control rats associated with a higher level of IFN-γ secretion. In contrast, Sudo et al. (2001) reported no significant difference in the splenocyte count and lymphocyte subsets of BALB/c mice fed with Biobran MGN-3 (0, 0.25%, and 0.5%) after 12 h of restrain stress. Significant reductions of splenocyte counts (*p* < 0.001) in all three groups compared to baseline were observed [67]. Hence, Biobran MGN-3 did not prevent the decrease in immune cell count due to psychological stress.

Kim et al. [45] also conducted a splenic lymphocyte proliferation assay with RBEP (10 and 100 μg/mL) and reported a 1.39- and 1.44-fold increase in cell proliferation levels in vitro relative to untreated control. However, the levels were lower compared to those produced with LPS and Con A. Similarly, Kang et al. [53] demonstrated that EFR could significantly increase splenocyte proliferation by 1.33 to 1.74 times (*p* < 0.05) compared to the control group at different concentrations in vitro (0.5 to 8 μg/mL). These results are consistent with the observation by Ghoneum and Abedi [65] that Biobran MGN-3 (0.1 mL with 10 mg/mL, i.p.) significantly increased splenic cellularity (145–192%, *p* < 0.025) in aged mice. Since the spleen cell population consists of all types of immune cells, further experiments with purified T and B cells by Chae et al. [55] showed that Biobran MGN-3 had minimal in vitro effects on splenic T cell proliferation and only induced B cell proliferation at a very high concentration (1000 μg/mL). Hence, the observations confirmed that while Biobran MGN-3 could induce immune cell proliferation in the spleen, it only indirectly affects T and B cell proliferation, likely by enhancing DC and macrophages’ antigen-presenting capability, as shown in Figure 5 earlier.

### 3.8. Mast Cells, Allergy, and Inflammation

The immune system is often considered a double-edged sword. While the system protects the host against infections and malignancies, an overreacting immunity could lead to detrimental consequences such as allergies, chronic inflammation, or autoimmune conditions. As RBAC has shown the capacity to stimulate different immune cells and enhance their activity, it is also essential to understand the role of RBAC during exaggerated immune responses in the host. Mast cells are immune cells widely distributed in host tissues and play a crucial role in the inflammatory response during infection and allergic reactions by releasing histamine and proinflammatory cytokines. Several studies were conducted to investigate the effects of RBAC on mast cells in various hypersensitivity scenarios, including anaphylaxis, asthma, and dermatitis, as shown in Table 8.

Kambayashi and Endo [84] studied the antiallergy effect of Biobran MGN-3 with a toluene diisocyanate (TDI)-induced asthmatic mice model. Female Balb/c mice sensitised with TDI were divided into four groups. Group A consumed Biobran MGN-3 (2 g/L dissolved in drinking water) one month before sensitisation and continued throughout the sensitisation and induction periods. Group B was administered Biobran MGN-3 (as prevention) one month before sensitisation and continued until the end of the sensitisation period. Group C was fed Biobran MGN-3 only during the TDI induction period (for symptom reduction). The last (D) was a control group that received no Biobran MGN-3. The effect of Biobran MGN-3 was evaluated by blood histamine concentration, eosinophil count in bronchoalveolar lavage fluid (BALF), TDI ear provocation test, and blood antibody titres (immunoglobulin [Ig] G1, IgG2a, and IgE) at the time of induction [84].

The study found the lowest blood histamine concentration in group A seven minutes after TDI induction at 2.5 ± 0.53 ng/mL. The levels were 4.2 ± 0.75 ng/mL in group B and 4.3 ± 0.78 ng/mL in group C [84]. All were significantly lower than the 6.4 ± 0.87 ng/mL of group D (*p* < 0.05). Furthermore, a 10- to 100-fold decrease in sensitivity compared to control was observed in the treatment groups with TDI ear provocation test at 0.01–10% concentrations. Biobran MGN-3 administration also significantly lowered the BALF eosinophil counts of the mice compared to the control. However, no effects on IgG and IgE production were found, suggesting that Biobran MGN-3 was a suppressor of mast cell activity during hypersensitivity [84].

In another study, Bae et al. [83] utilised the passive cutaneous anaphylaxis (PCA) titre to evaluate the antiallergy effect of Biobran MGN-3 in a murine model. All mice (Balb/c mice, *n* = 24) were sensitised with egg albumin as an allergen through subcutaneous injection before receiving Biobran MGN-3 or PSP (both at 1.5 mg/day p.o.) or saline (0.4 mL/day p.o.) for 14 days. PCA was carried out to induce an immediate dermal response caused by IgE and allergen reaction, showing as blue spots at the intradermal injection site. Biobran MGN-3-fed mice showed only 20 spots compared to 40 in the PSP group and 80 among the saline control mice (Bae et al., 2004). The plasma histamine content in the Biobran MGN-3-fed mice was also 25% lower than the control and PSP (6.5% lower) groups. Hence, in the presence of an allergen, Biobran MGN-3 downregulated the release of histamine from mast cells to reduce the inflammatory response [83].

Hoshino et al. [86] confirmed the in vitro effects of Biobran MGN-3 on mast cells. Using bone marrow-derived mast cells from BALB/c mice, the study reported that pretreatment with Biobran MGN-3 (0, 200, 500, 1000, 3000 μg/mL) for 30 min resulted in significant inhibition (*p* < 0.01) of β-hexosaminidase release (a marker for mast cell degranulation) after antigen stimulation in a dose-dependent manner. Notably, secretions of TNF-α and IL-4 by mast cells also demonstrated a significant dose-dependent reduction (*p* < 0.01) after antigen stimulation in mast cells pretreated with Biobran MGN-3. Follow-on investigations by Hoshino et al. [86] also revealed that Biobran MGN-3 pretreatment did not affect the early events of mast cell signal transduction (IgE binding, Ca2+ mobilisation, and phosphorylation of Akt) but significantly inhibited (*p* < 0.05) the distal event of membrane fusion between liposomes during degranulation activities mediated by soluble N-ethylmaleimide-sensitive factor attachment protein receptor-derived (SNARE) peptides. Moreover, suppression of the proinflammatory cytokine (TNF-α and IL-4) syntheses by Biobran MGN-3 pretreatment was shown to be through the nuclear factor kappa B (NF-κB) and mitogen-activated protein (MAP) kinase (extracellular signal-regulated kinase [ERK] and c-Jun N-terminal Kinase [JNK]) activation pathways [86].

Similarly, Kim et al. [85] confirmed that treatment with fermented SuperC3GHi bran (100 and 250 μg/mL) significantly reduced (*p* < 0.05) the release of β-hexosaminidase from RBL-2H3 mast cell line stimulated with the allergen sensitiser Compound 48/80. The β-hexosaminidase activity of mast cells was decreased by 25% (100 μg/mL) and 40% (250 μg/mL) compared to the control with no treatment. Analysis of histamine secretion also showed a similar trend, with a statistically significant 18% reduction (*p* < 0.05) recorded in mast cells treated with 100 μg/mL fermented SuperC3GHi bran and 43% with 250 μg/mL. Further experiments were carried out by Kim et al. [85] to assess the anti-inflammatory effect of fermented SuperC3GHi bran in an ear-oedema mice mode. Arachidonic acid (3%) was applied to the ears of ICR mice to induce inflammation via increased production of prostaglandins. Topical applications of the fermented SuperC3GHi bran were shown to reduce the inflammatory response by 35.6% compared to the controls. Such inhibition rate was less effective than indomethacin (68.5% reduction), a nonsteroidal anti-inflammatory drug, used as a positive control [85].

**Figure 6:**
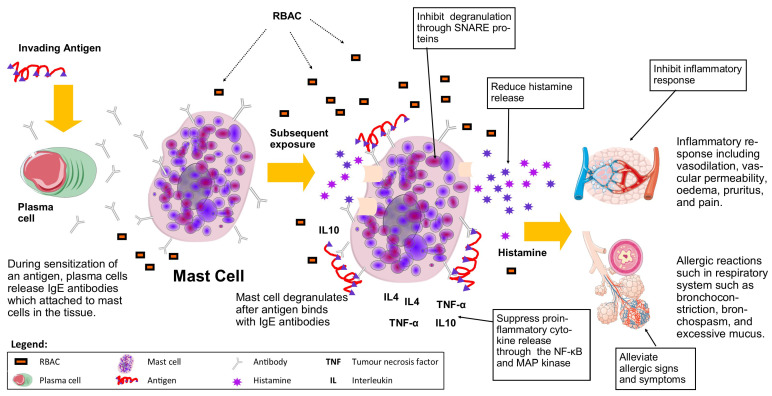
RBAC acts on mast cells to inhibit hypersensitivity and inflammatory responses during allergic reactions by inhibiting degranulation from releasing histamine and suppressing the production of proinflammatory cytokines (Boxed items are the effects of RBAC).

An [40] explored the antiallergy effects of RBEP on dinitrochlorobenzene (DNCB)-induced contact hypersensitivity in NC/Nga mice. The mice (*n* = 21) were divided equally into RBEP (250 mg/kg BW/day for 4 weeks), negative and positive control groups. Mice in RBEP and negative control groups were induced with dermatitis but not the positive controls. Significantly lower symptom scores (*p* < 0.05) in the RBEP group compared to the negative control group were recorded throughout the study during weekly assessments of skin lesions. Even though rising IgE concentrations in plasma and splenocytes were observed in the RBEP group compared to the positive controls at the end of the experiment, the levels were significantly lower (50% less) than those in the negative controls (*p* < 0.05). The RBEP group also recorded significantly lower IL-4 and IL-10 in both plasma and splenocytes compared to the negative controls (*p* < 0.05). The splenocyte IFN-γ and IFN-γ/IL-4 ratio of the RBEP group remained normal compared to the positive controls. In comparison, the negative control group registered significantly lower (*p* < 0.05) levels of IFN-γ and IFN-γ/IL-4 [40]. DNCB is a known allergen that causes dermatitis by forming immune complex deposits (IgG1 conjugates) that activate the complement system, contributing to the development of hypersensitivity reactions [87]. Hence, RBEP alleviated the allergic reaction to DNCB by preventing the cytokine production imbalance favouring Th2 responses (humoural immunity of B cells). Figure 6 summarises the role of mast cells in an allergic reaction and the effects of RBAC on mast cells to alleviate the allergic signs and symptoms and inhibit inflammatory responses.

### 3.9. Vascular Endothelial Growth Factor and Antiangiogenic Effects

Angiogenesis, the formation of new blood vessels, is a complex process involving the migration, growth, and differentiation of endothelial cells to line the interior wall of blood vessels. Chemical signalling proteins control the process, and among them is vascular endothelial growth factor (VEGF), a proangiogenic growth factor [88]. VEGF also plays a critical role in the immune environment. With VEGF receptors expressed in DC, macrophages, and T lymphocytes, the functioning of these immune cells, including differentiation, maturation, and chemotaxis, is influenced by VEGF [89]. VEGF is also a mediator synthesised during the activation of mast cells that promotes inflammation and vascular permeability [90]. Overexpression of VEGF is associated with tumour growth and autoimmune diseases such as rheumatoid arthritis, systemic sclerosis, and multiple sclerosis [91,92]. Insufficient VEGF signalling, on the other hand, can lead to a decrease in vascular function, which drives physiological aging across multiple organ systems [93]. Hence, modulating the VEGF signalling pathway may favour health and prevent aging.

Zhu et al. [94] investigated the effects of Biobran MGN-3 on VEGF-induced angiogenesis in vitro. Human umbilical vein endothelial cells (HUVECs) and human dermal fibroblasts were co-cultured in the presence or absence of VEGF (10 ng/mL) with or without Biobran MGN-3 (0.3, 1, or 3 mg/mL). Biobran MGN-3 was found to inhibit tube formation in HUVECs under the microscope. Quantitative analysis using an image analyser on the area, length, joint, and path of tubes exhibited dose-dependent inhibitory effects with higher concentrations (1 and 3 mg/mL) negated the VEGF-induced tube formation to the level of passive control [94]. Furthermore, Biobran MGN-3 also significantly suppressed (*p* < 0.01) the VEGF-induced proliferation and migration of HUVECs in a dose-dependent manner. Through western blot analysis, the anti-angiogenic effects of Biobran MGN-3 (3 mg/mL) significantly decreased the VEGF-induced phosphorylation of VEGF receptor 2 and the downstream signalling proteins Akt, ERK1/2, and p38 in HUVECs co-cultured with VEGF (10 ng/mL). In comparison, Biobran MGN-3 (3 mg/mL) treatment alone caused no significant change in the expression of these proteins [94]. However, it remains unclear how Biobran MGN-3 may affect the angiogenic process under different concentrations of VEGF.

### 3.10. Effects on Circulating Cytokines

As discussed in the previous sections, many preclinical studies have demonstrated RBAC’s ability to modulate cytokine production for priming macrophages, augmenting NKC activity, inducing DC maturation, promoting T and B cell proliferation, and downregulating mast cells in allergic responses. Cytokines are potent immune modulators often produced in a cascade, with one cytokine stimulating the target cells to produce additional cytokines [95]. Cytokines can be pro- or anti-inflammatory. Proinflammatory cytokines, the key ones being IL-1β, IL-6, and TNF-α, are involved in the upregulation of inflammatory reactions, resulting in pathological signs of pain, heat, redness, swelling, and loss of function [95,96]. Major anti-inflammatory cytokines are the IL-1 receptor antagonist, IL-4, IL-10, IL-11, and IL-13, and they attenuate inflammation to allow tissue repair and regeneration [97]. Dysregulation of cytokine production can lead to an inflammatory state which increases the risk of chronic diseases, of which aging is an associative factor [98,99]. As such, circulating cytokine levels, especially proinflammatory cytokines, have been suggested as a potential biomarker for inflammatory diseases and aging [100,101,102]. Table 9 shows a list of clinical studies evaluating RBAC’s effects on circulating cytokines in healthy adults.

Ali et al. [69] investigated the effects of Biobran MGN-3 (1 and 3 g/day for 60 days) on plasma cytokines and growth factors in healthy individuals (*n* = 20). A total of 12 cytokines and growth factors (IL-2, IL-4, IL-6, IL-8, IL-10, IL-1α, IL-1β, IFN-γ, TNF-α, monocyte chemoattractant protein-1, VEGF, and epidermal growth factor [EGF]) were tested at baseline, 1 week, 30 days, and 60 days. Except for IL-4 and IL-8, the study found significant effects (*p* < 0.05) for time but not between groups (1 g/day vs. 3 g/day) and group-by-time interaction. IFN-γ, TNF-α, IL-1α, IL-1β, IL-8, and IL-10, and EGF peaked at 30 days and returned to the baseline level at 60 days. Hence, this study showed that the effects of Biobran MGN-3 in healthy adults were not purely proinflammatory nor anti-inflammatory but immunomodulatory over time [69].

Choi et al. [26] studied the serum cytokine level of healthy volunteers (*n* = 80) randomly assigned to take either RBEP or placebo (3 g/day) for 8 weeks. IFN-γ, TNF-α, IL-2, IL-4, IL-10, and IL-12 were tested at baseline, week 4 and week 8. The study showed that RBEP significantly increased serum IFN-γ compared to placebo at week 8 (35.56 ± 17.66 vs. 27.04 ± 12.51 pg/mL, *p* < 0.05), whereas no significant differences were detected for other cytokines. Moreover, there was no significant difference in reported adverse events among the participants in both RBEP and placebo groups. IFN-γ responses are implicated in the host defence against intracellular pathogens, immune modulation, inflammatory processes associated with tissue damage and tumour immunosurveillance [103]. The increased serum IFN-γ, a proinflammatory cytokine, could indicate enhanced immunity against infections in healthy participants as IFN-γ is produced predominantly by NKC during the innate immune response and by CD4+ and CD8+ T lymphocytes once adaptive immunity develops [104]. Hence, IFN-γ production was suggested as a supportive marker of NKC activity in certain malignancies [105]. In this case, the increased IFN-γ could indirectly indicate elevated NKC activity in the healthy participants in the short term, even though the same study did not detect a significant difference in NKC cytotoxic activity between RBAC and placebo group [26].

Nevertheless, combining available evidence from both human studies [26,69], healthy individuals may likely experience fluctuations in circulating cytokine levels after taking RBAC due to increased immune activity. The effect may be short-term and may eventually stabilise. However, the immune cells have been primed with RBAC and thus could mount a more potent immune response should there be an actual infection based on the premise of trained immunity, a function of the innate defence mechanism [106].

### 3.11. Effects among Older Adults

With RBAC’s immunomodulatory capacity, how RBAC could impact immunosenescence is another area of interest. The effects of Biobran MGN-3 on geriatric participants with reduced immune functions were investigated in three studies shown in Table 10.

The field of psychoneuroimmunology strongly suggests a reciprocal and complex interaction between mind and body with perceived health-related quality of life (QoL) as a result of and directly affects cognitive functioning, the immune system and disease process [107]. Hence, improving the immunity in aging adults was hypothesised to enhance the QoL. Elsaid et al. [108] evaluated the effects of Biobran MGN-3 on the QoL of generally healthy older adults (≥56 years old). The study recruited 60 participants (40 males and 20 females) and randomly assigned them to either Biobran MGN-3 (250 mg/day) or placebo groups with a supplementation duration of 3 months. QoL was assessed with the validated SF-12v2 health questionnaire before and after the intervention. The study found that Biobran MGN-3 significantly improved the QoL of older adults after 3 months compared to baseline, whereas no significant differences in QoL scores were detected in the placebo group. Between-group comparisons showed that Biobran MGN-3 had significantly improved both the physical (*p* < 0.037) and mental (*p* < 0.012) component summary scores and the specific component scores of role physical (*p* < 0.002), bodily pain (*p* < 0.006), vitality (*p* < 0.000), and social functioning (*p* < 0.001). Hence, Elsaid et al. [108] concluded that a low dose of Biobran MGN-3 (250 mg/day) over 3 months could improve the QoL of older adults.

**Table 10:** The immunomodulating effects of RBAC on older adults with evidence from human clinical trials.

No	RBAC (dose)	Participants	Key Findings	Author (Year)
1.	Biobran MGN-3 (500 mg/day for 2 weeks with cross-over)	Older adults (age 75–90, *n* = 36)	The mean total symptom score for CCS during the control period (0.1535 ± 0.38591) was significantly higher than that of the Biobran MGN-3 treatment period (0.0491 ± 0.09083, *p* = 0.0426). Biobran MGN-3 significantly prevented the decrease in T lymphocyte blastogenesis based on PHA mitogen test compared to the control.	Tazawa et al. (2003) [71]
2.	Biobran MGN-3 (250 mg/day for 3 months)	Older adults (age ≥ 56, *n* = 60, 30 in each group)	Biobran MGN-3 significantly improved the QoL of older adults compared to baseline. Compared to placebo, Biobran MGN-3 significantly improved both the physical (*p* < 0.037) and mental (*p* < 0.012) component summary scores and the specific component scores of role physical (*p* < 0.002), bodily pain (*p* < 0.006), vitality (*p* < 0.000), and social functioning (*p* < 0.001).	Elsaid et al. (2020) [108]
3.	Biobran MGN-3 (500 mg/day for 3 months)	Older adults (age ≥ 56, *n* = 80, 40 in each group)	The Biobran MGN-3 group had significantly lower ILI incidents than the placebo group (5% vs. 22.5%, *p* = 0.048). The risk of ILI infection in the Biobran MGN-3 group was estimated to be 18.2% (95% CI: 3.9–48.9), which was significantly lower (*p* < 0.05) than the 55.1% (95% CI: 43.4–66.3) for the placebo group.	Elsaid et al. (2021) [74]

Abbreviations: CCS, common cold syndrome; CI, confidence interval; ILI, influenza-like illnesses; PHA, phytohemagglutinin; QoL, quality of life; RBAC, rice bran arabinoxylan compound.

Another RCT by Elsaid et al. [74] focused on the effects of Biobran MGN-3 on strengthening the innate immunity of older adults by assessing the incidents of influenza-like illnesses (ILI). Immunosenescence is known to amplify susceptibility to viral infections, such as influenza, even with vaccination. Older people also have a greater risk of more severe ILI, leading to severe respiratory symptoms, systemic complications, hospitalization, and death [109,110]. Hence, Biobran MGN-3, as an antioxidant and immunomodulator, could reduce the risk of contracting ILI while lowering the symptom severity in those infected.

Eighty generally healthy older adults (≥56 years old, male-to-female ratio: 1 to 1) enrolled in the study by Elsaid et al. [74]. Participants were randomly allocated to take either 500 mg/day of Biobran MGN-3 or placebo powder for 3 months, with the incidence of ILI infection monitored during the trial. The study found significantly lower ILI incidents (*p* = 0.048) in the Biobran MGN-3 group (5%, 2/40) compared to the placebo group (22.5%, 9/40). The risk of ILI infection in the Biobran MGN-3 group was estimated to be 18.2% (95% confidence interval [CI]: 3.9–48.9), which was significantly lower (*p* < 0.05) than the 55.1% (95% CI: 43.4-66.3) for the placebo group. Hence, the data suggest that Biobran MGN-3 could have prophylactic effects against ILI among older adults [74].

Similarly, an earlier study by Tazawa et al. [71] among older adults (age 70–95) under institutional elderly care also confirmed the effects of Biobran MGN-3 in reducing the severity of common cold symptoms (CCS). In this cross-over study, participants were taking 500 mg/day Biobran MGN-3 (treatment) and a water-soluble unmodified rice bran powder of similar taste (control) for 2 weeks each in random order with 2-week washout in between (total 6 weeks). During the 42 days trial period, participants were monitored for CCS (fever, headache, malaise, chills, cough, sputum, running/stuffy nose, sore throat and chest pain) by care personnel at the facility who were blinded to their intervention allocation. Each symptom was scored based on a severity grade of 0 (no symptom) to 3 (severe) [71].

Results from participants (*n* = 36, mean age 84 ± 7.2) who completed the trial of Tazawa et al. [71] showed that there was no significant difference in the number of participants for whom CCS was observed (*n* = 15 each). The mean duration of CCS during the control period was double that of the treatment period (2.6 ± 5.94 vs. 1.2 ± 2.20 days) [71]. However, the difference did not reach statistical significance (*p* = 0.2721). Nonetheless, the mean total symptom score for CCS during the control period (0.1535 ± 0.38591) was significantly higher than that of the treatment period (0.0491 ± 0.09083, *p* = 0.0426). Furthermore, a decrease in T lymphocyte blastogenesis by the phytohemagglutinin mitogens test was detected in both periods. However, the reduction during the Biobran MGN3 treatment period was significantly lower than in the control period (*p* < 0.05) [71]. As such, the results supported the hypothesis that Biobran MGN-3 could reduce the severity of CCS among older people by improving immunity.

### 3.12. Safety and Adverse Events

RBAC is generally considered safe to consume as a derivative of edible rice bran. In vitro tests also showed that products like RBEP and Biobran MGN-3 are not cytotoxic and did not cause cell damage [40,56]. The safety data reported by BioBran Research Foundation [111] showed the single-dose toxicity of Biobran MGN-3 in Wistar rats to be above 36 g/kg (LD_50_). Repeat-dose toxicity studies found the No-Observed-Adverse-Effect Level (NOAEL) of Biobran MGN-3 to be 1301 mg/kg/day for male SD rats, 1562 mg/kg/day for female SD rats, and 200 mg/kg/day for Beagle dogs [111]. Among the human studies, no adverse events due to RBAC were reported. Moreover, safety measurements based on laboratory tests during clinical trials also showed that RBAC did not adversely affect the kidney and liver system [26,63,69,71,73,74,108].

## 4. Discussion

This review identified a group of RBAC products that are polysaccharide derivatives from defatted rice bran denatured with the mycelia enzymes of *L. edodes*. The active ingredients of Biobran MGN-3, the most studied among all, comprise heteropolysaccharides with arabinoxylan as its primary structure (∼36%) plus galactan and glucan [22]. However, the natural sugar composition of different RBAC products or even the subfraction of the same product can differ drastically, demonstrating the heterogeneity in the macromolecular structures of polysaccharides [22,23,24,25,26]. The results from such chemical composition analysis should be interpreted with caution as it is a considerable challenge to fully anatomise the convoluted levels of complexity within the polysaccharide derivatives [112]. Regardless of the diversity in sugar composition, all RBAC products have demonstrated promising immunological effects. Above all, RBAC is highly water-soluble [22], can interact with the immune cells at Peyer’s patches after digestion [45] and is absorbed through the intestinal tract to exert biological activities [43]. However, the extent of RBAC’s bioavailability remained unclear with the lack of any pharmacokinetic study [113]. A recent preliminary investigation by Schupfer et al. [114] found that RBAC consumption as a dietary supplement could potentially modulate the gut microbiome of some healthy individuals. Hence, it is likely for RBAC to be only partially absorbed in the digestive tract, with the unabsorbed portion serving as prebiotics to the gut microbiota. Given that microbiome-immunity crosstalk is known to impact human health and disease [115], the ability to modulate gut microbiome could be an alternative route of RBAC influencing the host immune system. This topic warrants further investigation.

One of RBAC’s health and anti-aging effects could be its antioxidant properties. RBAC possesses strong free radical scavenging activities against O_2_^•−^ and •OH and the antioxidant capacity of RBAC is deemed to be on par or even excel that of broccoli [39,40]. Treatment with RBAC in mice has also demostrated systemic improvement in endogenous antioxidant enzymes and reduction of oxidative stress [41]. Damage from oxidative stress is suggested as a hallmark of aging and crucial in developing age-related diseases [116,117]. An imbalance in antioxidant defence against ROS could lead to prolonged inflammation and pathophysiological development of debilitating chronic illnesses, such as cardiovascular diseases, diabetes, cancer, or neurodegenerative conditions [29]. As an antioxidant, RBAC can protect host tissue against the collateral damage caused by free radicals. Experiments with different disease models also demonstrated that RBAC could protect the liver against ROS generated during biotransformation and metabolism of xenobiotics [118,119]. Using a streptozotocin-induced Alzheimer’s disease mice model, Ghoneum and El Sayed [120] also showed that RBAC could prevent amyloid-beta-induced apoptosis in the brain through its antioxidant properties. Additionally, RBAC could also protect against oxidative damage caused by environmental or clinical γ-irradiation exposure in vitro and in vivo [121,122,123]. Hence, the antioxidant capacity of RBAC is highly beneficial in preventing chronic conditions resulting from oxidative stress.

The immune cells are particularly susceptible to oxidative stress as the high percentage of polyunsaturated fatty acids in their plasma membranes could be degraded due to free radical chain reactions under the robust production of ROS [28,124]. As antioxidants, RBAC can protect host tissue against collateral damage by free radicals during phagocytosis while upregulating the activities of phagocytes, including monocytes, macrophages, and neutrophils [22,23,53,54,55,56]. Specifically, RBAC has been shown to increase the phagocytosis rate of macrophages [23,54,55,56,57] while enhancing the lysosomal enzyme [24,25,45,52,53] and NO production in macrophages [22,23,53,55] to destroy invading pathogens. RBAC can also stimulate cytokine secretion in macrophages, especially TNF-α and IL-6, to initiate inflammation during infection [23,53,55,56,58] and activate autophagy-related proteins (Beclin-1, Atg5, Atg12, Atg16L) for clearing cellular debris and regulating inflammatory responses [23]. Decreased phagocytosis and reduced autophagy in macrophages are salient characteristics of chronic inflammation in old age, or ‘inflammaging’, as pointed out by De Maeyer and Chambers [125]. To this end, RBAC could help to prevent the inflammaging state in advancing years.

RBAC’s ability to enhance NKC activity has been extensively studied in vitro, in vivo, and in clinical trials [26,58,63,64,65,66,67,68,69,70,71,73,74]. Available evidence suggests that RBAC is a potent inducer of NKC cytotoxicity against aberrant cells due to viral infection and tumorigenesis [58,63,64,65,66,67,68], achieved through increased CD69 and CD25 surface markers [58,64]. RBAC also increases cytokine production in NKC, notably IFN-γ, TNF-α, and IL-2 [63,64,67]. The primary role of IFN-γ is to restrict viral propagation and tumour progression [126,127], whereas TNF-α and IL-2 further induce functional maturation in NKC and promote the growth and development of other immune cells, yielding a more robust immune response [128,129]. The results from human clinical trials show that the effects of RBAC on NKC are immunomodulatory rather than immunostimulatory in healthy individuals with no risk of overstimulation [26,69,73,74]. RBAC’s effects on NKC are more prominent in individuals with weakened immunity, either acquired (e.g., chemical exposure) or due to old age [70,73,74].

Aging can lead to changes in the NKC population, cytotoxicity and proliferation, as well as impairment in the ratio of different phenotypes [130,131]. Such age-associated changes in NKC function have broad implications for health, such as the slow resolution of inflammatory responses and increased incidence of infections [132]. The benefits of RBAC’s improved NKC function are evident in human trials showing reduced ILI incidence and severity of CCS in geriatric volunteers, thus preventing older adults from severe respiratory and systemic complications [71,72,74]. Influenza-linked morbidity and mortality are high among older adults and represent a significant healthcare burden. For example, the influenza-associated excess respiratory hospitalisation and mortality rates for older adults aged ≥ 75 years in Australia were 302.95 (95% CI: 144.71–461.19) and 35.11 (95% CI: 19.93–50.29) per 100,000 population, respectively [133]. Such high hospitalisation and mortality rates are sustained even with the widespread availability and extensive coverage of influenza vaccines (68.5% among older adults aged ≥ 75 years) [134]. RBAC could be a safe and effective preventative strategy to reduce the ILI disease burden among the older population while improving their QoL [71,74,108].

Besides strengthening the immunosurveillance through phagocytic and NKC activity, RBAC is also capable of activating adaptive immunity through DC by inducing maturation of surface markers (upregulation of CD83, CD86, and DEC-205) [79,80,81], reducing endocytic activity [79], stimulating cytokine production (IL-1β, IL-6, IL-10, TNF-α, IL-12p40, IL-12p70, IL-2 and IL-29) [80,81], and enhancing the capacity to activate T lymphocytes (CD4+ and CD8+ T cells) [80,81]. Consequently, T and B cell proliferation is commonly observed in lymphoid organs such as the spleen due to DC antigen presentation [45,53,65,67,83]. Notably, RBAC appears to not directly affect the proliferation of T and B cells and the cytotoxicity of natural killer T cells [55].

Another indicator of RBAC being an immunomodulator instead of purely a stimulator is the ability to downregulate the inflammatory response during allergic hypersensitivity reactions [40,83,84,85,86]. Current research has recognised mast cells as the common effectors of allergic reactions and autoimmune pathways [135,136]. By acting on mast cells, RBAC inhibits degranulation mediated by SNARE proteins [86], which lower histamine secretion after antigen stimulation [83,84,85]. RBAC also reduces the production of proinflammatory cytokines (TNF-α and IL-4) synthesised by mast cells through the NF-κB and MAP kinase (ERK & JNK) activation pathways [40,86]. Thus, RBAC could also play a role in the prevention of allergic conditions (asthma, eczema, hay fever, etc.) and autoimmune disorders (rheumatism, inflammatory bowel disease, type 1 diabetes, etc.) with which aging is a leading risk factor [137].

More research is required to understand RBAC’s biological effects and potential benefits as an immunoceutical for health and aging, especially after long-term consumption. Particularly, RBAC is a source of plant-based PAMP molecules, which could be promising therapeutic tools for treating malignant tumours [44,138] and a molecular agent for trained immunity to increase the immune response against potential infection in healthy adults [106]. However, the authors could not find any RBAC structures registered on PubChem during the review process, mainly due to limited molecular chemistry research in this area. The lack of understanding of the chemical composition of RBAC and the likely PAMP configurations that lead to its biological activities has hampered its clinical application as a immunomodular [138]. Hence, future research needs to clarify the active ingredients further and the molecular targets responsible for immunomodulating activities leading to the standardisation of the quality and quantity of the active compound for clinical use.

Moreover, questions remain regarding RBAC’s bioavailability, pharmacodynamics, and pharmacokinetics of the complex heteropolysaccharides, which are also essential for future research on RBAC’s therapeutic applications. Regarding biological activity, the discovery of RBAC effects on VEGF-induced angiogenesis represents a novel front for further understanding how RBAC may affect the haematopoiesis of immune cells and inflammatory responses and whether RBAC acts as a stimulatory or modulatory agent [94]. A possible direction of future investigation will be to understand how RBAC could affect angiogenesis in the tumour microenvironment and how the angiogenesis effect of RBAC could prevent neoplasm in healthy cells. Additionally, more translational research is urgently needed to assess the prophylactic effects of RBAC as an immunoceutical for the aging population, especially against infections and immune-related disorders, with human studies of larger sample sizes and cohort studies with long follow-up periods. Such translational efforts could potentially help to improve QoL for the world’s aging population with RBAC as a widely assessable plant-based compound.

## 5. Conclusions

RBAC is a safe and effective neutraceutical for improving immune health, especially for aging adults with weakened immunity. For a generally healthy person, a low dosage of 500 mg to 1 g per day for 2 to 3 months may boost immunity through immunosurveillance improvement and lowering oxidative stress. There is no risk of overstimulating the immune system as RBAC is a modulator that can augment immune response while downregulating hyperactive allergic reactions. The expected benefits can be prevention against infections, such as influenza, and lowering the risk of chronic inflammatory conditions.

## Figures and Tables

**Table 1:** Comparing the natural sugar composition of different RBAC products or fractions with the unit of measure in mol %.

RBAC	Sample	Rha	Fuc	Ara	Xyl	Man	Glc	Gal
Biobran	Fr II	1.0	0.2	23.0	15.1	2.0	48.1	10.7
MGN-3	Fr I-4	1.1	0.4	22.5	19.4	4.7	26.7	25.2
	Fr I-5	2.7	0.7	22.2	14.8	3.5	32.2	23.9
	Fr I-6 *	7.6	0.6	22.2	13.7	2.7	30.2	23.0
	Fr III	1.9	5.9	15.7	7.5	1.3	30.1	37.8
	Fr IV	-	-	2.7	1.7	2.4	89.2	4.0
RBEP	1	4.0	1.7	21.8	9.0	22.9	22.0	18.6
	2	NR	NR	Trace	22.25	NR	11.71	Trace
BPP		2.7	0.8	NR	3.0 **		18.2	9.1
BPRBE		NR	NR	NR	0.59	10.8	0.09	55.1
NPRBE ***		NR	NR	NR	-	-	0.17	-

Abbreviation: Ara, Arabinose; BPP, Bioprocessed polysaccharide (STR Biotech); BPRBE, Bioprocessed rice bran extract (STR Biotech); Fuc, Fucose; Gal, Galactose; Glc, Glucose; Man, Mannose; NPRBE, Non-bioprocessed rice bran extract (STR Biotech); NR, not reported; RBEP, Rice bran exo-biopolymer (Erom); Rha, rhamnose; Xyl, xylose. Note: * II-6 was the subfraction of Biobran MGN-3 that demonstrated the highest immunomodulating activity in vitro. ** This number is based on xylose with mannose combined. *** NPRBE is not an RBAC but serves as a control for comparison only.

**Table 2:** A summary of the antioxidant capacity of RBAC.

No	RBAC (Dose)	Assay/Model	Key Findings	Author (Year)
1 .	Biobran MGN-3 (20, 2.0, and 0.2 mg/mL)	Spin trapping, Fenton reaction, and ultraviolet reaction.	The ROS scavenging rates were dose-dependent against O_2_^•−^ and •OH. The S fraction Biobran MGN-3 (<3000 molecules) was the most excellent in inhibiting •OH caused by O_2_^•−^ and ultraviolet irradiation.	Tazawa et al. (2000) [39]
2 .	RBEP (1 g/15 mL [ORAC], 1 g/20 mL [TAS])	ORAC and TAS.	Both fat- and water-soluble RBEP had much higher ORAC values (446 and 326 μM TE/g) than lipophilic and hydrophilic extracts from raw broccoli, broccoli seed, and sprout. RBEP had a higher TAS measurement (0.4 mmol/100 g) than raw broccoli (0.18) and broccoli seeds (0.38) but not broccoli sprouts (0.56).	An (2011) [40]
3 .	Biobran MGN-3 (25 mg/kg BW i.p. 6x/day for 25 days, 19x total)	Female Swiss albino mice (*n* = 10 per group)	Mice treated with Biobran MGN-3 had significantly higher GSH and GPx in the liver versus the control group. Biobran MGN-3 also upregulated the GPx, and CAT mRNA (*p* < 0.05) expression compared to the control mice.	Noaman et al. (2008) [41]

Abbreviations: CAT, catalase; GPx, glutathione peroxidase; GSH, glutathione; i.p. intraperitoneal injection; mRNA, messenger ribonucleic acid; ORAC, oxygen radical antioxidant capacity; RBAC, rice bran arabinoxylan compound; RBEP, rice bran exo-biopolymer; ROS, reactive oxygen species; TAS, Total Antioxidant Status; TE, trolox equivalent.

**Table 3:** A summary of the effects of RBAC on macrophages based on available in vitro and in vivo evidence.

No	RBAC (Dose)	Cell Type	Key Findings	Author (Year)
**A. Enhance lysosomal enzyme production**				
1.	RBEP (10 and 100 μg/mL)	P-M∅ (in vitro)	RBEP showed the highest stimulating activity in both concentrations compared to 8 other rice bran extracts cultured with different fungi. The levels were similar to LPS at 10 μg/mL and higher at 100 μg/mL.	Yu et al. (2004) [25]
2.	RBEP (10 and 100 μg/mL)	P-M∅ (in vitro)	Both doses (10 and 100 μg/mL) elicited more than twice the control activity level, similar to the levels of LPS under the same amount.	Kim et al. (2005) [45]
3.	RBEP (50 and 250 mg/kg BW p.o.)	P-M∅ (in vivo)	Dose-dependent (1.41- and 1.44-fold) increases in lysosomal enzyme activity were recorded compared to the control in P-M∅ extracted from mice fed with REBP for 5 days.	Kim et al. (2005) [45]
4.	CFP (250 mg/kg BW p.o. for 4 weeks)	P-M∅ (in vivo)	Lysosomal enzyme activity was 104.60 ± 10.97% relative to control. The secondary fermented products either increased (CFP-S, 115.21 ± 18.94%) or decreased activities (CFP-L, 97.99 ± 16.79%). The activities of CFP and CFP-L were significantly enhanced (*p* < 0.05) in the presence of LPS but reduced in the case of CFP-S.	Kim et al. (2011) [52]
5.	BPP (10 and 100 μg/mL)	P-M∅ (in vitro)	BPP could increase the lysosomal enzyme activity by ∼5.4-fold at 10 μg/mL and ∼2.5-fold at 100 μg/mL, compared to the polysaccharides of *L. edodes* alone. At 100 μg/mL, BPP also exhibited a higher activity than LPS.	Kim et al. 2013 [24]
6.	EFR (0.5, 1, 2, 4 and 8 μg/mL)	P-M∅ (in vitro)	Significant increases in enzyme activity relative to the control were observed (134.9–142.2% at different concentrations, *p* < 0.05). Such levels are at par with the 135.8% increase demonstrated by LPS.	Kang et al. (2022) [53]
**B. Increase phagocytosis rate**				
7.	Biobran MGN-3 (1, 10 and 100 μg/mL)	U937, RAW264.7, and P-M∅ (in vitro)	Biobran MGN-3 enhanced the rate of attachment and phagocytosis of yeast in a dose-dependent manner, with responsiveness varied across cell types.	Ghoneum & Matsuura (2004) [54]
8.	Biobran MGN-3 (1.5 mg/L p.o. for 10 days)	P-M∅ (in vivo)	The phagocytic activity of P-M∅ treated with Biobran MGN-3 (69%) was comparable with those with PSP (65%) treatment against Candida parapsilosis, and both were significantly higher (*p* < 0.05) than the control (47%).	Chae et al. (2004) [55]
9.	Biobran MGN-3 (100 μg/mL)	Human PBL (in vitro)	Biobran MGN-3 (100 μg/mL) significantly enhanced (*p* < 0.01) the phagocytosis of *Escherichia coli* by monocytes (110%) and neutrophils (400%) compared to the control. Increased oxidative burst with hydrogen peroxide in the presence of *E. coli* was observed.	Ghoneum et al. (2008) [56]
10.	BPP (1, 10 and 100 μg/mL)	RAW 264.7 (in vitro)	Bacterial uptake rates against *Salmonella typhimurium* increased by 1.4-, 2.4-, and 3.5-fold in a dose-dependent manner compared to untreated macrophages. A greater intracellular bacteria presence after 2 h compared to the control, but the bacterial count decreased after 4 and 8 h relative to the control.	Kim et al. (2014) [57]
11.	BPP(10 mg/kg BW p.o. & i.p.)	P-M∅ (in vivo)	Pretreatment with BPP (p.o. and i.p.) for 14 days significantly reduced (*p* < 0.05) *S. typhimurium* count in the peritoneal cavity of the infected mice.	Kim et al. (2014) [57]
12.	BPRBE (1, 10 and 100 μg/mL)	RAW264.7 (in vitro)	Dose-dependent increases in phagocytotic rates against *S. typhimurium* at 1.3-, 2.3-, and 3.4-fold compared to the control were observed.	Kim et al. (2018) [23]
**C. Induce NO production**				
13.	Biobran MGN-3 (1, 10, 100 and 1000 μg/mL)	P-M∅ (in vitro)	Crude Biobran MGN-3 induced NO production in general, but different fractions of Biobran MGN-3 yielded NO differently at a dose-dependent rate.	Miura et al. (2004) [22]
14.	Biobran MGN-3 (1, 10, 100 and 1000 μg/mL)	RAW264.7 (in vitro)	Biobran MGN-3 and PSP were observed to increase NO production compared to the control, but Biobran MGN-3 appeared less effective than PSP.	Chae et al. (2004) [55]
15.	BPRBE (1, 10 and 100 μg/mL)	RAW264.7 (in vitro)	NO production significantly increased relative to both passive control and non-bioprocessed rice bran extract for all doses and LPS for doses 10 μg/mL. Increased iNOS (∼1.9 fold) expression was detected.	Kim et al. (2018) [23]
16.	EFR (0.5, 1, 2, 4 and 8 μg/mL)	RAW264.7 (in vitro)	A dose-dependent significant increase (*p* < 0.05) in NO production was observed compared to control with an associated increase in iNOS expression. The level of NO production in 8 μg/mL of EFR was comparable to that of 1 μg/mL of LPS.	Kang et al. (2022) [53]
**D. Influence cytokine secretion**				
17.	Biobran MGN-3 (1000 μg/mL)	RAW264.7 (in vitro)	Biobran MGN-3 induced higher levels of IL-6 and TNF-α production than the control but much less than the levels influenced by PSP and LPS.	Chae et al. (2004) [55]
18.	Biobran MGN-3 (1, 10, 100 and 1000 μg/mL)	U937 (in vitro)	Dose-dependently increases TNF-α, IL-6, IL-8, and IL-10 production with significant (*p* < 0.01) differences observed at 10 μg/mL compared to control.	Ghoneum et al. (2008) [56]
19.	Biobran MGN-3 (0.01, 0.1, 1, 10 and 100 μg/mL)	Human macrophages (in vitro)	Only higher levels of Biobran MGN-3 (10 μg/mL and above) resulted in the release of IL-8, IL-6 and TNF-α but significantly lower than LPS.	Pérez-Martínez et al. (2015) [58]
20.	BPRBE (100 μg/mL)	RAW264.7 (in vitro)	Treatment markedly induced (*p* < 0.05) IFN-β production in the *Salmonella*-infected macrophage cells via the IFR3 pathway.	Kim et al. (2018) [23]
21.	EFR (0.5, 1, 2, 4 and 8 μg/mL)	RAW264.7 (in vitro)	Significant increases (*p* < 0.05) in the secretion of IL-1β, IL-10, IL-6, and TNF-α compared to the control in a dose-dependent manner.	Kang et al. (2022) [53]
**E. Cross-presenting of antigens**				
22.	Biobran MGN-3 (1000 μg/mL)	RAW264.7 (in vitro)	Biobran MGN-3 increased the MHC-II expression compared to the control but at a level lower than PSP, LPS, and IFN-γ.	Chae et al. (2004) [55]
23.	BPP (100 μg/mL)	RAW 264.7 (in vitro)	BPP-treated cells showed dendrite-like morphological changes, reaching up to 3.6-, 10.3-, and 12.4-fold changes after 2, 4, and 8 h of incubation.	Kim et al. (2014) [57]
**F. Activate autophagy**				
24.	BPRBE (100 μg/mL)	RAW264.7 (in vitro)	BPRBE treatment was found to upregulate the expression of the autophagy-related proteins (Beclin-1, Atg5, Atg12, Atg16L) regardless of bacterial infection.	Kim et al. (2018) [23]

Abbreviations: BPP, bioprocessed polysaccharide; BPRBE, bioprocessed rice bran extract; BW, body weight; CFP, crude fermented polysaccharide; CFP-L, CFP extract fermented with *Lactobacillus gasseri*; CFP-S, CFP extract fermented with *Saccharomyces cerevisiae*; EFR, Erom’s fermented rice bran; IFN, interferon; IFR, interferon regulatory pathway; IL, interleukin; iNOS, inducible nitric oxide synthase; i.p., intraperitoneal injection; LPS, lipopolysaccharide; MHC-II, major histocompatibility complex class II; NO, nitric oxide; PBL, peripheral blood lymphocytes; P-M∅, peritoneal macrophage; p.o., per oral; PSP, polysaccharide-peptide; RBAC, rice bran arabinoxylan compound; RBEP, rice bran exo-biopolymer; TNF, tumour necrosis factor.

**Table 4:** A summary of the effects of RBAC on NKC activity based on available in vitro and in vivo evidence.

No	RBAC (Dose)	Model	Key Findings	Author (Year)
**A. Enhance NKC cytotoxicity (chromium assay)**				
1.	Biobran MGN-3 (25 and 100 μg/mL)	PBL from healthy human donors (*n* = 6)	Biobran MGN-3 significantly increased (*p* < 0.001) NKC cytotoxicity in vitro in a dose-dependent manner (130% at 25 μg/mL vs. 150% at 100 μg/mL).	Ghoneum (1998) [63]
2.	Biobran MGN-3 (500 μg/mL)	PBL and purified NKC from healthy human donors (*n* = 25)	Significant increases in NKC cytotoxicity in both PBL (*p* < 0.001) and purified NKC (*p* < 0.01) by Biobran MGN-3. The NKC augmentation effect of Biobran MGN-3 was enhanced by co-culturing with IL-2 (500 U/mL) in PBL but not in purified NKC.	Ghoneum & Jewett (2000) [64]
3.	Biobran MGN-3 (10 mg/kg BW, i.p. or p.o. daily)	Aged C57BL/6 and C3H female mice	Biobran MGN-3 i.p. significantly increased peritoneal NKC activity (4.8–6-fold, *p* < 0.01) from day 2 to 14 but not splenic NKC activity. However, oral feeding of Biobran MGN-3 elicited a 200% increase (*p* < 0.01) in splenic NKC activity but not peritoneal NKC activity.	Ghoneum & Abedi (2004) [65]
4.	Biobran MGN-3 (25 and 100 μg/mL)	Splenic lymphocytes from aged C57BL/6 mice	In vitro NKC activity exhibited a 4-fold increase (*p* < 0.01) over the control after culturing with Biobran MGN-3.	Ghoneum & Abedi (2004) [65]
5.	Biobran MGN-3 (100 μg/mL i.m. daily)	Young Swiss albino mice	The treated mice showed a significant increase in splenic NKC activity (27.1 LUs vs. 8.3 LUs, *p* < 0.01) compared to the control after 14 days.	Badr El-Din et al. (2008) [66]
6.	Biobran MGN-3 (250 mg/day p.o.)	Male Lewis rats	Rats fed with Biobran MGN-3 as a dietary supplement showed significantly enhanced splenic NKC activity (*p* < 0.045) compared to rats fed with only a control diet after two weeks.	Giese et al. (2008) [67]
7.	RBEP (50 mg/kg or 250 mg/kg, p.o. daily)	Male ICR mice	RBEP was shown to enhance the splenic NKC activity significantly after 5 days, reporting 32.8 LUs (50 mg/kg, *p* < 0.05) and 46.3 LUs (250 mg/kg, *p* < 0.01) compared to the 22.8 LUs of the controls.	Kim et al. (2007) [68]
8.	Biobran MGN-3 (100 μg/mL)	NKC isolated from PBL of healthy donors	Significant increases (*p* < 0.05) in NKC cytotoxicity against 7 different tested cell lines compared to resting NKC with overnight in vitro stimulation of Biobran MGN-3. However, the levels were lower than IL-5 (10 ng/mL) stimulated NKC. Biobran MGN-3 also synergistically enhanced the stimulatory effect (*p* < 0.05) of low-dose IL-2 (40 IU/mL) on NKC cytotoxicity level to that obtained with 1000 IU/mL of IL-2.	Pérez-Martínez et al. (2015) [58]
**B. Elevate NKC activity (other indicators)**				
9.	Biobran MGN-3 (10 mg/kg BW, i.p. daily)	C57BL/6 mice (*n* = 3)	A remarkable increase in BLT-esterase activity (*p* < 0.01) and granularity after 5 days of i.p. was detected. The binding capacity of peritoneal NKC also significantly increased with higher percentage conjugates (26% vs. 13%, *p* < 0.01) compared to the control.	Ghoneum & Abedi (2004) [65]
10.	Biobran MGN-3 (100 μg/mL, i.m. daily)	Young Swiss albino mice	A two-fold increase (27.5% vs. 14%, *p* < 0.01) in the splenic NKC binding capacity in the percentage of conjugate formation to target cells was detected after 14 days.	Badr El-Din et al. (2008) [66]
**C. Increase cytokine production**				
11.	Biobran MGN-3 (25, 50 and 100 μg/mL)	PBL from healthy human donors (*n* = 6)	Biobran MGN-3 also increased IFN-γ production significantly (*p* < 0.001), showing 340, 390, and 580 pg/mL of IFN-γ production at 25, 50, and 100 μg/mL, respectively, compared to <100 pg/mL in control.	Ghoneum (1998) [63]
12.	Biobran MGN-3 (1, 10, 100 and 1000 μg/mL)	PBL and purified NKC from healthy human donors (*n* = 25 for TNF-α, *n* = 14 for IFN-γ)	Biobran MGN-3 at higher concentrations (100 and 1000 μg/mL) significantly increased TNF-α (*p* < 0.001) and IFN-γ (*p* < 0.03) secretions in PBL with variations across samples. IL-2 + Biobran MGN-3 further enhanced TNF-α secretion by PBL but not purified NKC. A synergistic effect of IL-2 + Biobran MGN-3 on IFN-γ production by purified NKC was detected.	Ghoneum & Jewett (2000) [64]
13.	Biobran MGN-3 (250 mg/day p.o.)	Male Lewis rats (*n* = 24)	Significant elevations of IL-2 (*p* < 0.05) and IFN-γ (*p* < 0.08) production after 14 days in splenocytes of rats fed with Biobran MGN-3 compared to control.	Giese et al. (2008) [67]
**D. Expression of cell surface markers**				
14.	Biobran MGN-3 (100 and 1000 μg/mL)	PBL from healthy human donors (*n* = 25)	Upregulation of CD69, CD54, and CD25 was observed in NKC after Biobran MGN-3 treatment at levels comparable to IL-2-stimulated NKC.	Ghoneum & Jewett (2000) [64]
15.	Biobran MGN-3 (100 μg/mL)	NKC isolated from PBL of healthy donors	NKC stimulated with Biobran MGN-3 showed increased expression of CD69 and CD25 by 3.1-fold and 3.2-fold over resting NKC, respectively.	Pérez-Martínez et al. (2015) [58]

Abbreviations: CD, cluster of differentiation; IFN, interferon; IL, interleukin; i.m. intramuscular injection; i.p., intraperitoneal injection; LU, lysis unit; NKC, natural killer cells; PBL, peripheral blood lymphocytes; p.o., per oral; RBAC, rice bran arabinoxylan compound; RBEP, rice bran exo-biopolymer; TNF, tumour necrosis factor.

**Table 5:** The effects of RBAC on NKC activity of healthy adults with evidence from human clinical trials.

No	RBAC (Dose)	Participants	Key Findings	Author (Year)
1.	Biobran MGN-3 (15, 30, 45 mg/kg BW/day for 2 months)	Healthy adults (*n* = 24, 8 in each group)	Significantly enhanced NKC cytotoxicity was achieved at 1 week (∼3.1-fold, *p* < 0.001) for 30 and 45 mg/kg doses and after 1 month (2-fold) for 15 mg/kg. Peak response was achieved after 2 months (∼5-fold) for all doses and returned to baseline after 1 month of discontinuing treatment. The percentage of conjugate formations increased significantly over baseline (38.5% vs. 9.4%, *p* < 0.005) after 1 month at 45 mg/kg dose.	Ghoneum (1998) [63]
2.	Biobran MGN-3 (15 mg/kg BW/day for 4 months)	Adults with workplace chemical exposure (*n* = 11)	The participants had low levels of NKC activity (10.2 ± 4.2 LUs) at baseline, but treatment with MGN-3 increased NKC activity 4- and 7-fold at 2 and 4 months, respectively. T and B cell functions were 130–150% higher than base line values.	Ghoneum (1999) [70]
3.	Biobran MGN-3 (500 mg/day for 2 weeks with cross-over)	Older adults (age 75–90, *n* = 31)	There was no significant difference in NKC activity before and after the study and across intervention periods. A secondary analysis showed that those with low NKC activity at baseline (*n* = 12, ≤30%) had higher activity during treatment compared to the control (↑ 34.1% vs. 15.6%).	Tazawa et al. (2003) [71] Maeda et al. (2004) [72]
4.	Biobran MGN-3 (1, 3 g/day for 60 days)	Healthy adults (*n* = 20, 10 in each group)	A significant effect of time (*p* = 0.001) on the NKC activity was detected but not for the group and the interaction of group and time. Total NKC activity peaked at 1 week and was significantly higher than the values at other time points (0, 48 h, 30 and 60 days).	Ali et al. (2012) [69]
5.	RBEP (3 g/day or placebo for 8 weeks)	Healthy adults (*n* = 80, 40 in each group)	No significant effect of RBEP supplementation on NKC activity over placebo at all time points (0, 4, 8 weeks)	Choi et al. (2014) [26]
6.	Biobran MGN-3 (500 mg/day for 1 month)	Older adults (age ≥ 56, *n* = 12, 6 in each group)	The median CD107a expression (NKC activity) in the Biobran MGN-3 group significantly increased from 60.5% to 83.0% after 1 month (*p* < 0.046), with no significant differences detected in the placebo group.	Elsaid et al. (2018) [73]
7.	Biobran MGN-3 (500 mg/day for 3 month)	Older adults (age ≥ 56, *n* = 12, 6 in each group)	Mean NKC CD107a expression significantly increased (*p* = 0.004) from 49.5 ± 10.4% to 75.2 ± 6.6% for the Biobran MGN-3 group compared to an insignificant difference in the placebo group (45.3 ± 12 vs. 50.8 ± 19.5).	Elsaid et al. (2021) [74]

Abbreviations: BW, body weight; CD, cluster of differentiation; LU, lytic unit; NKC, natural killer cells; RBAC, rice bran arabinoxylan compound.

**Table 6:** A summary of the effects of RBAC on monocyte-derived DC based on available in vitro evidence.

No	RBAC (Dose)	Cell Type	Key Findings	Author (Year)
**A. Induce DC maturation**				
1.	Biobran MGN-3 (10, 100, 400 and 1000 μg/mL)	Monocytes isolated from buffy coats of healthy donors.	BioBran MGN3 downregulated the CD14 and CD1a on the surface of iDC while markedly increasing CD80, CD83, and CD86 expressions. Also, BioBran MGN-3 decreased CD11c surface antigen and increased CD123.	Cholujova et al. (2009) [79]
2.	Biobran MGN-3 (5, 10 and 20 μg/mL)	Monocyte-derived DCs	Flow cytometry analysis showed a dose-dependent upregulation in CD83 and CD86 surface markers similar to the effects of LPS on DC.	Ghoneum & Agrawal (2011) [80]
3.	Biobran MGN-3 (20 and 40 μg/mL)	Monocyte-derived DCs	BioBran MGN3 showed signs of maturation with increased DEC-205 expression for antigen-presenting in a dose-dependent manner.	Ghoneum & Agrawal (2014) [81]
**B. Reduce endocytic activity**				
4.	Biobran MGN-3 (100, 400 and 1000 μg/mL)	Monocytes isolated from buffy coats of healthy donors.	BioBran MGN3 reduced the endocytic activity of iDC from 73% to 27.7% (100 μg/mL), 17.7% (400 μg/mL), and 14.4% (1000 μg/mL) under different concentrations, approaching the levels of mDC.	Cholujova et al. (2009) [79]
**C. Stimulate cytokine secretion**				
5.	Biobran MGN-3 (5, 10 and 20 μg/mL)	Monocyte-derived DCs	BioBran MGN3 treated DC produced significantly higher levels (*p* < 0.05) of IL-1β, IL-6, IL-10, TNF-α, IL-12p40, IL-12p70, and IL-2, compared to untreated DC.	Ghoneum & Agrawal (2011) [80]
6.	Biobran MGN-3 (10 and 20 μg/mL)	Monocyte-derived DCs	Treatment of 20 μg/mL of BioBran MGN3 significantly increased IL-29 production in DC (*p* < 0.05) compared to the control. Increased secretion of IFN-α and -β were also detected but did not achieve statistical significance.	Ghoneum & Agrawal (2014) [81]
**D. Enhance the capacity to activate T lymphocytes**				
7.	Biobran MGN-3 (10 μg/mL)	Monocyte-derived DCs	Co-culturing of DC pretreated with 10 μg/mL BioBran MGN3 with allogeneic CD4+ T cells increased the proliferation by 1.4 fold and CD25 marker expression by 1.3 fold, compared to those cultured with untreated DC.	Ghoneum & Agrawal (2011) [80]
8.	Biobran MGN-3 (20 μg/mL)	Monocyte-derived DCs	Culturing the activated DC (by BioBran MGN3) with purified, allogeneic CD8+T cells resulted in significantly higher levels of granzyme B-positive CD8+ T cells (*p* < 0.05) with increased cytotoxicity against tumour cell targets (*p* < 0.05), compared to control.	Ghoneum & Agrawal (2014) [81]

Abbreviations: CD, cluster of differentiation; DC, dendritic cells; iDC, immature dendritic cells; IFN, interferon; IL, interleukin; LPS, lipopolysaccharide; mDC, matured dendritic cells; RBAC, rice bran arabinoxylan compound.

**Table 7:** A summary of the effects of RBAC on splenic cell proliferation with a focus on T and B lymphocyte activity based on available in vitro and in vivo evidence.

No	RBAC (Dose)	Cell Type	Key Findings	Author (Year)
**A. Increase plague and rosette formation**				
1.	Biobran MGN-3 (1.5 mg/day p.o. for 10 days)	Splenocytes from BALB/c mice	Rosette formation due to higher antibody production increased by 30% over control (*p* < 0.005). Splenocyte plague formation was also highest in the Biobran MGN-3 group, with a 14% increase over the control group.	Bae et al. (2004) [83] Chae et al. (2004) [55]
**B. Enhance splenocyte proliferative activity**				
2.	Biobran MGN-3 (1, 10, 100, 1000 μg/mL)	Splenocytes from BALB/c mice	Biobran MGN-3 induced splenocyte proliferation in a dose-dependent manner at a much lower level than LPS, Con A and PSP. Biobran MGN-3 also induced IFN-γ secretion like LPS in splenocytes.	Bae et al. (2004) [83]
3.	Biobran MGN-3 (10 mg/kg BW/day, i.p. or p.o. for 2, 5, & 14 days)	Splenocytes from aged C57BL/6 and C3H female mice	Biobran MGN-3 significantly increased splenic cellularity in both strains of mice by 145 to 192% (*p* < 0.025) compared to the control.	Ghoneum & Abedi (2004) [65]
4.	RBEP (10, 100 μg/mL)	Splenocytes from ICR mice	A 1.39- to 1.44-fold increase in cell proliferation levels in vitro relative to untreated control was detected. The levels were lower compared to that produced with LPS and Con A.	Kim et al. (2005) [45]
5.	Biobran MGN-3 (0.25 g/day for 14 days)	Splenocytes from male Lewis rats (*n* = 24)	Biobran MGN3-fed rats showed a significantly higher splenocyte proliferative activity against the superantigen TSST-1 compared to control rats and associated with a higher level of IFN-γ secretion.	Giese et al. (2008) [67]
6.	EFR (0.5 to 8 μg/mL)	Splenocytes from C57/BL6 mice	EFR could significantly increase in vitro splenocyte proliferation by 1.33 to 1.74 times (*p* < 0.05) compared to the control.	Kang et al. (2022) [53]
**C. Purified T and B cell proliferative activity**				
7.	Biobran MGN-3 (1, 10, 100, 1000 μg/mL)	Purified T and B cells isolated from Splenocytes of BALB/c mice	Biobran MGN-3 had minimal in vitro effects on splenic T cell proliferation and only induced B cell proliferation at a very high concentration (1000 μg/mL).	Chae et al. (2004) [55]

Abbreviations: Con A, concanavalin A; EFR, Erom’s fermented rice bran; IFN, interferon; LPS, lipopolysaccharide; RBAC, rice bran arabinoxylan compound; RBEP, rice bran exo-biopolymer; TSST-1, toxic shock syndrome toxin-1.

**Table 8:** A summary of the effects of RBAC on mast cells and hypersensitive immune responses based on available in vitro and in vivo evidence.

No	RBAC (Dose)	Model	Key Findings	Author (Year)
**A. Alleviate allergic signs and symptoms**				
1.	Biobran MGN-3 (2 g/L drinking water for 1 month)	TDI-induced asthmatic Balb/c mice	A 10- to 100-fold decrease in sensitivity compared to control was observed with Biobran MGN-3 treatment under TDI ear provocation test at 0.01–10% concentrations. Biobran MGN-3 also significantly lowered the eosinophil counts in the BALF of asthmatic mice. No effects on IgG and IgE production were found.	Kambayashi & Endo (2002) [84]
2.	Biobran MGN-3 (1.5 mg/day p.o. for 14 days)	PCA-titre in Balb/c mice (*n* = 24)	Biobran MGN-3-fed mice showed a lower allergic dermal response (20 spots) after PCA induction compared to 40 in the PSP group and 80 among the saline control mice.	Bae et al. (2004) [83]
3.	RBEP (250 mg/kg BW/day for 4 weeks)	DNCB-induced contact dermatitis in NC/Nga mice (*n* = 21)	Significantly lower symptom scores (*p* < 0.05) in the RBEP group compared to the negative control group. Rising IgE concentrations in plasma and splenocytes were observed in the RBEP group but at levels significantly lower (50% less) than those in the negative controls (*p* < 0.05).	An (2011) [40]
**B. Reduce histamine release**				
4.	Biobran MGN-3 (2 g/L drinking water for 1 month)	TDI-induced asthmatic Balb/c mice	Significantly lower (*p* < 0.05) plasma histamine concentrations were detected in all Biobran MGN-3 treated mice compared to the control.	Kambayashi & Endo (2002) [84]
5.	Biobran MGN-3 (1.5 mg/day p.o. for 14 days)	PCA-titre in Balb/c mice (*n* = 24)	The plasma histamine content in the Biobran MGN-3-fed mice was also 25% lower than in the control and PSP (6.5% lower) groups.	Bae et al. (2004) [83]
6.	Fermented SuperC3GHi bran (100 and 250 μg/mL)	RBL-2H3 mast cell line	In vitro histamine secretion analysis showed a statistically significant 18% reduction (*p* < 0.05) recorded in stimulated mast cells treated with 100 μg/mL fermented SuperC3GHi bran and 43% with 250 μg/mL.	Kim et al. (2011) [85]
**C. Inhibit mast cell degranulation**				
7.	Biobran MGN-3 (200, 500, 1000, 3000 μg/mL)	Bone marrow-derived mast cells from BALB/c mice	Pretreatment with Biobran MGN-3 for 30 min resulted in significant inhibition (*p* < 0.01) of β-hexosaminidase release after antigen stimulation in a dose-dependent manner. Biobran MGN-3 significantly inhibited (*p* < 0.05) the degranulation activities mediated by SNARE proteins.	Hoshino et al. (2010) [86]
8.	Fermented SuperC3GHi bran (100 and 250 μg/mL)	RBL-2H3 mast cell line	Treatment with fermented SuperC3GHi bran significantly reduced (*p* < 0.05) the release of β-hexosaminidase in antigen-stimulated mast cells by 25% (100 μg/mL) and 40% (250 μg/mL) compared to the controls.	Kim et al. (2011) [85]
**D. Regulate proinflammatory cytokine production**				
9.	Biobran MGN-3 (200, 500, 1000, 3000 μg/mL)	Bone marrow-derived mast cells from BALB/c mice	Biobran MGN-3 suppressed the proinflammatory cytokines (TNF-α and IL-4) syntheses potentially through the NF-κB and MAP kinase (ERK & JNK) activation pathways.	Hoshino et al. (2010) [86]
10.	RBEP (250 mg/kg BW/day for 4 weeks)	DNCB-induced contact dermatitis in NC/Nga mice (*n* = 21)	RBEP significantly lowered IL-4 and IL-10 in plasma and splenocytes compared to the negative controls (*p* < 0.05). The splenocyte IFN-γ and IFN-γ/IL-4 ratio of the RBEP group remained normal, whereas the negative control group registered significantly lower (*p* < 0.05) levels of IFN-γ and IFN-γ/IL-4 ratio.	An (2011) [40]
**E. Inhibit mast cell degranulation**				
11.	Fermented SuperC3GHi bran (100 and 250 μg/mL)	AA-induced ear-oedema in ICR mice	The inflammatory rates (% increase in ear thickness) were 23.5% (control), 14.6% (SuperC3GHi), and 8.5% (indomethacin). SuperC3GHi bran reduced inflammatory response by 35.6% compared to controls, less effective than the 68.5% of indomethacin, a nonsteroidal anti-inflammatory drug.	Kim et al. (2011) [85]

Abbreviations: AA, arachidonic acid; BALF, bronchoalveolar lavage fluid; BW, body weight; DNCB, dinitrochlorobenzene; ERK, extracellular signal-regulated kinase; IFN, interferon; Ig, immunoglobulin; IL, interleukin; JNK, c-Jun N-terminal Kinase; MAP, mitogen-activated protein; NF-kB, nuclear factor kappa B; PCA, Passive cutaneous anaphylaxis; PSP, polysaccharide-peptide; RBAC, rice bran arabinoxylan compound; RBEP, rice bran exo-biopolymer; SNARE, soluble N-ethylmaleimide-sensitive factor attachment protein receptor; TDI, toluene diisocyanate; top, topical application.

**Table 9:** The effects of RBAC on circulating cytokines in healthy adults with evidence from human clinical trials.

No	RBAC (Dose)	Participants	Key Findings	Author (Year)
1.	Biobran MGN-3 (1 or 3 g/day for 60 days)	Healthy adults (*n* = 20, 10 in each group)	The study found significant effects (*p* < 0.05) over time (baseline, 1 week, 30 days, and 60 days) for IL-2, IL-6, IL-10, IL-1α, IL-1β, IFN-γ, TNF-α, MCP-1, VEGF, EGF. IFN-γ, TNF-α, IL-1α, IL-1β, IL-8, and IL-10, and EGF peaked at 30 days and returned to the baseline level at 60 days.	Ali et al. (2012) [69]
2.	RBEP (3 g/day or placebo for 8 weeks)	Healthy adults (*n* = 80, 40 in each group)	RBEP significantly increased serum IFN-γ compared to placebo at week 8 (35.56 ± 17.66 vs. 27.04 ± 12.51 pg/mL, *p* < 0.05), whereas no significant differences were detected for other cytokines (TNF-α, IL-2, IL-4, IL-10, and IL-12).	Choi et al. (2004) [26]

Abbreviations: EGF, epidermal growth factor; IFN, interferon; IL, interleukin; MCP, monocyte chemoattractant protein; RBAC, rice bran arabinoxylan compound; TNF, tumour necrosis factor; VEGF, vascular endothelial growth factor.

## Data Availability

The data that support the findings of this study are available from the corresponding authors (S.C.P.) upon reasonable request.

## Supplementary Materials

The following supporting information can be downloaded at: https://www.mdpi.com/article/10.3390/molecules28176313/s1, Supplementary S1: Table S1. The characteristics of all included articles in alphabetic order of the first author’s name; Supplementary S2: Full citation of all included articles in APA 7th format.

## Abbreviations

The following abbreviations are used in this manuscript:

BALF	bronchoalveolar lavage fluid
BPP	Bioprocessed polysaccharides
BPRBE	Bioprocessed rice bran extract
BW	Body weight
CCS	Common cold symptoms
CFP	Fermented black rice bran
CMM	Cytokine maturation mix
CON A	Concanavalin A
CI	Confidence interval
DC	Dendritic cell
DNA	Deoxyribonucleic acid
DNAM	DNAX Accessory Molecule
DNCB	Dinitrochlorobenzene
EFR	Erom’s fermented rice bran
ERK	Extracellular signal-regulated kinase
EGF	Epidermal growth factor [EGF]
HUVECs	Human umbilical vein endothelial cells
ICAM-1	intercellular adhesion molecule-1
Ig	immunoglobulin
IFN	Interferon
IL	Interleukin
ILI	influanza-like illnesses
iDC	immatured dendritic cell
iNOS	Inducible nitric oxide synthase
i.p.	Intraperitoneal injection
JNK	c-Jun N-terminal kinase
LPS	lipopolysaccharide
LU	Lytic unit
MAP	Mitogen-activated protein
matDC	Matured dendritic cell
MHC-II	Major histocompatibility complex class II
mRNA	messenger Ribonucleic acid
NCR	Nuclear receptor coregulators
NF-κB	nuclear factor kappa B
NKC	Natural kill cell
NKG2D	Natural killer group 2D
NO	Nitric oxide
ORAC	Oxygen radical antioxidant capacity
PAMP	Pathogenic-associated molecular pattern
PBL	Peripheral blood lymphocytes
PBMC	Peripheral blood-derived mononuclear cells
PCA	Passive cutaneous anaphylaxis
P-M∅	Peritoneal macrophage
p.o.	Per oral
PSP	Polysaccharide-peptide
QoL	Quality of life
RBAC	Rice bran arabinoxylan compound
RBEP	Rice bran exo-biopolymer
RCT	Randomised controlled trial
ROS	Reactive oxygen species
RT-PCR	Reverse transcriptase polymerase chain reaction
SNARE	Soluble N-ethylmaleimide-sensitive factor attachment protein receptor
TAS	Total antioxidant status
TDI	Toluene diisocyanate
TLR	Toll-like receptor
TNF	Tumour necrosis factor
VEGF	Vascular endothelial growth factor
