# Supplementary material for: Modified Rice Bran Arabinoxylan by Lentinus edodes Mycelial Enzyme as an Immunoceutical for Health and Aging—A Comprehensive Literature Review
Source: Molecules. 2023 Aug 29;28(17):6313. doi: 10.3390/molecules28176313 (PMC10488663; doi:10.3390/molecules28176313)
Supplement: Supplementary file 1 [file molecules-28-06313-s001.zip › molecules-2507092-supplementary.pdf]

Supplementary S1. The included studies

Table S1. The characteristics of all included articles in alphabetic order of the first author's name.

#	Article ID	Type	RBAC	Source	Design	Condition	Biological Action
1	Ali et al. (2012)	Article	Biobran MGN-3	Daiwa	Human RCT	Healthy	Immunomodulation (NK cells, cytokines)
2	An (2011)	Thesis	RBEP/EFR	Erom	Animal, cell, chemical	Healthy, allergy	Cytotoxicity, antiallergy, antioxidant
3	Bae et al. (2004)	Article	Biobran MGN-3	Daiwa	Animal	Healthy, allergy	Immunomodulation (macrophages, splenocytes), antiallergy
4	Badr El-Din et al. (2008)	Article	Biobran MGN-3	Daiwa	Animal	Healthy	Immunomodulation (NK cells)
5	Chae et al. (2004)	Article	Biobran MGN-3	Daiwa	Cell	Healthy	Immunomodulation (splenocytes, macrophages, T & B cells, cytokines)
6	Choi et al. (2014)	Article	RBEP/EFR	Erom	Human RCT	Healthy	Immunomodulation (NK cells, macrophages)
7	Cholujova et al. (2009)	Article	Biobran MGN-3	Daiwa	Cell	Healthy	Immunomodulation (Dendritic cells)
8	Elsaid et al. (2018)	Article	Biobran MGN-3	Daiwa	Human RCT	Geriatric	Immunomodulation (NK cells, NK T cells)
9	Elsaid et al. (2020)	Article	Biobran MGN-3	Daiwa	Human RCT	Geriatric	Psychoneuroimmuno-modulation
10	Elsaid et al. (2021)	Article	Biobran MGN-3	Daiwa	Human RCT	Geriatric, cold / flu	Flu prevention
11	Endo and Kanbayashi (2003)	Short paper	Biobran MGN-3	Daiwa	Animal	Healthy	Bioavailability, allergy (antibodies)
12	Ghoneum and Abedi (2004)	Article	Biobran MGN-3	Daiwa	Animal, cell	Aging	Immunomodulation (Macrophages, NK cells)
13	Ghoneum and Agrawal (2011)	Article	Biobran MGN-3	Daiwa	Cell	Healthy	Immunomodulation (Dendritic cells, T cells, cytokines)
14	Ghoneum and Agrawal (2014)	Article	Biobran MGN-3	Daiwa	Cell	Healthy	Immunomodulation (Dendritic cells, T cells, cytokines)
15	Ghoneum and Jewett (2000)	Article	Biobran MGN-3	Daiwa	Cell	Healthy	Immunomodulation (NK cells, cytokines)
16	Ghoneum and Matsuura (2004)	Article	Biobran MGN-3	Daiwa	Cell	Healthy	Immunomodulation (Macrophages, cytokines)
17	Ghoneum (1998)	Article	Biobran MGN-3	Daiwa	Human Bef-Aft	Healthy	Immunomodulation (NK cells)
18	Ghoneum (1999)	Abstract	Biobran MGN-3	Daiwa	Human Bef-Aft	Chemical exposure	Immunomodulation (NK cells, T & B cells)
19	Ghoneum et al. (2008)	Article	Biobran MGN-3	Daiwa	Cell	Healthy	Antibacterial, phagocytic cells (monocytes, neutrophils)
20	Giese et al. (2008)	Article	Biobran MGN-3	Daiwa	Animal	Healthy	Immunomodulation (NK cells, cytokines)
21	Hoshino et al. (2010)	Article	Biobran MGN-3	Daiwa	Cell	Healthy	Antiinflammation (Mast cells, cytokines, antibodies)
22	Kambayashi and Endo (2002)	Abstract	Biobran MGN-3	Daiwa	Animal	Allergy	Antiallergy, antiinflammation (antibodies)
23	Kang et al. (2022)	Article	RBEP/EFR	Erom	Cell	Healthy	Immunomodulation (Splenocytes, macrophages, cytokines)
24	Kim D.J. et al. (2011a)	Article	Fermented SuperC3GHi bran	Erom	Animal, cell	Healthy, allergy	Antiallergy (mast cells), antiinflammation

25	Kim D.J. et al. (2011b)	Article	Fermented black rice bran	STR Biotech	Animal	Healthy	Immunomodulation (macrophages)
26	Kim H.Y. et al. (2005)	Article	RBEP/EFR	Erom	Animal, cell	Healthy	Immunomodulation (Peyer's patch cells, macrophages)
27	Kim S.P. et al. (2013)	Article	BPP/FF	STR Biotech	Cell	Healthy	Immunomodulation (macrophages)
28	Kim S.P. et al. (2014)	Article	BPP/FF	STR Biotech	Animal, cell	Healthy, bacterial infection	Immunomodulation (macrophages), antibacterial
29	Kim S.P. et al. (2018)	Article	BPRBE	STR Biotech	Cell	Healthy	Immunomodulation (macrophages, cytokines)
30	Miura et al. (2004/2013)	Article	Biobran MGN-3	Daiwa	Chemical, cell	Healthy	Immunomodulation (macrophages, cytokines)
31	Noaman et al. (2008)	Article	Biobran MGN-3	Daiwa	Animal	Healthy	Antioxidant
32	Pérez-Martínez et al. (2015)	Article	Biobran MGN-3	Daiwa	Cell	Healthy	Immunomodulation (macrophages, splenocytes, cytokines)
33	Sudo et al. (2001)	Article	Biobran MGN-3	Daiwa	Animal	Endotoxemia	Antiinflammation
34	Tazawa et al. (2000)	Article	Biobran MGN-3	Daiwa	Chemical	Reactive oxygen species	Antioxidant
35	Tazawa et al. (2003)	Article	Biobran MGN-3	Daiwa	Human RCT	Geriatric, cold / flu	Immunomodulation, flu prevention
36	Yu et al. (2004)	Article	RBEP/EFR	Erom	Cell	Healthy	Immunomodulation (macrophages)
37	Zhu et al. (2017)	Article	Biobran MGN-3	Daiwa	Cell	Healthy	Angiogenesis (growth factor)

Abbreviations: Bef-Aft, before and after study**;** BPP/FF, bioprocessed polysaccharide or fermented black rice bran; BPRBE, bioprocessed rice bran extract; NK, natural killer; RBEP/EFR**,** rice bran bio-exopolymer or Erom's fermented rice bran**;** RCT, randomised controlled trial.

Supplementary S2. Full citation of all included articles in APA 7th format

References

Ali, K. H., Melillo, A. B., Leonard, S. M., Asthana, D., Woolger, J. M., Wolfson, A. H., McDaniel, H., & Lewis, J. E. (2012). An open-label, randomized clinical trial to assess the immunomodulatory activity of a novel oligosaccharide compound in healthy adults. *Functional Foods in Health and Disease, 2*(7), 265.

An, S. Y. (2011). *Immune-enhance and anti-tumor effect of exo-biopolymer extract from submerged culture of Lentinus edodes with rice bran* [Doctoral Thesis, Korea University]. Seoul, Republic of Korea.

Badr El-Din, N. K., Noaman, E., & Ghoneum, M. (2008). In vivo tumor inhibitory effects of nutritional rice bran supplement MGN-3/Biobran on Ehrlich carcinoma-bearing mice. *Nutrition and Cancer, 60*(2), 235-244. https://doi.org/10.1080/01635580701627285

Bae, M. J., Lee, S. T., Chae, S. Y., Shin, S. H., Kwon, S. H., Park, M. H., Song, M. Y., & Hwang, S. J. (2004). The effects of the arabinoxylane and the polysaccharide peptide (PSP) on the antiallergy, anticancer. *Journal of the Korean Society of Food Science and Nutrition, 33*(3), 469-474. https://doi.org/10.3746/jkfn.2004.33.3.469

Chae, S. Y., Shin, S. H., Bae, M. J., Park, M. H., Song, M. K., Hwang, S. J., & Yee, S. T. (2004). Effect of arabinoxylane and PSP on activation of immune cells. *Journal of the Korean Society of Food Science and Nutrition, 33*(2), 278-286. https://doi.org/10.3746/jkfn.2004.33.2.278

Choi, J. Y., Paik, D. J., Kwon, D. Y., & Park, Y. (2014). Dietary supplementation with rice bran fermented with Lentinus edodes increases interferon-γ activity without causing adverse effects: a randomized, double-blind, placebo-controlled, parallel-group study. *Nutrition Journal, 13*, 35. https://doi.org/10.1186/1475-2891-13-35

Cholujova, D., Jakubikova, J., & Sedlak, J. (2009). BioBran-augmented maturation of human monocyte-derived dendritic cells. *Neoplasma, 56*(2), 89-95. https://doi.org/10.4149/neo_2009_02_89

Elsaid, A. F., Agrawal, S., Agrawal, A., & Ghoneum, M. (2021). Dietary supplementation with Biobran/MGN-3 increases innate resistance and reduces the incidence of influenza-like illnesses in elderly subjects: a randomized, double-blind, placebo-controlled pilot clinical trial. *Nutrients, 13*(11). https://doi.org/10.3390/nu13114133

Elsaid, A. F., Fahmi, R. M., Shaheen, M., & Ghoneum, M. (2020). The enhancing effects of Biobran/MGN-3, an arabinoxylan rice bran, on healthy old adults' health-related quality of life: a randomized, double-blind, placebo-controlled clinical trial. *Quality of Life Research, 29*(2), 357-367. https://doi.org/10.1007/s11136-019-02286-7

Elsaid, A. F., Shaheen, M., & Ghoneum, M. (2018). Biobran/MGN-3, an arabinoxylan rice bran, enhances NK cell activity in geriatric subjects: A randomized, double-blind, placebo-controlled clinical trial. *Experimental and Therapeutic Medicine, 15*(3), 2313-2320. https://doi.org/10.3892/etm.2018.5713

Endo, Y., & Kanbayashi, H. (2003). Modified rice bran beneficial for weight loss of mice as a major and acute adverse effect of cisplatin. *Pharmacology and Toxicology, 92*(6), 300-303. https://doi.org/10.1034/j.1600-0773.2003.920608.x

Ghoneum, M. (1998). Enhancement of human natural killer cell activity by modified arabinoxylane from rice bran (MGN-3). *International Journal of Immunotherapy, 14*(2), 89-99.

Ghoneum, M. (1999, Dec 11-13). *Immunostimulation and cancer prevention* [Abstract]. 7th International Congress on Anti-Aging & Biomedical Technologies, Las Vegas, NV, USA.

Ghoneum, M., & Abedi, S. (2004). Enhancement of natural killer cell activity of aged mice by modified arabinoxylan rice bran (MGN-3/Biobran). *Journal of Pharmacy and Pharmacology, 56*(12), 1581-1588. https://doi.org/10.1211/0022357044922

Ghoneum, M., & Agrawal, S. (2011). Activation of human monocyte-derived dendritic cells in vitro by the biological response modifier arabinoxylan rice bran (MGN-3/Biobran). *International Journal of Immunopathology and Pharmacology, 24*(4), 941-948. https://doi.org/10.1177/039463201102400412

Ghoneum, M., & Agrawal, S. (2014). MGN-3/biobran enhances generation of cytotoxic CD8+ T cells via upregulation of DEC-205 expression on dendritic cells. *International Journal of Immunopathology and Pharmacology, 27*(4), 523-530. https://doi.org/10.1177/039463201402700408

Ghoneum, M., & Jewett, A. (2000). Production of tumor necrosis factor-alpha and interferon-gamma from human peripheral blood lymphocytes by MGN-3, a modified arabinoxylan from rice bran, and its synergy with interleukin-2 in vitro. *Cancer Detection and Prevention, 24*(4), 314-324.

Ghoneum, M., & Matsuura, M. (2004). Augmentation of macrophage phagocytosis by modified arabinoxylan rice bran (MGN-3/biobran). *International Journal of Immunopathology and Pharmacology, 17*(3), 283-292. https://doi.org/10.1177/039463200401700308

Ghoneum, M., Matsuura, M., & Gollapudi, S. (2008). Modified arabinoxylan rice bran (MGN-3/Biobran) enhances intracellular killing of microbes by human phagocytic cells in vitro. *International Journal of Immunopathology and Pharmacology, 21*(1), 87-95. https://doi.org/10.1177/039463200802100110

Giese, S., Sabell, G. R., & Coussons-Read, M. (2008). Impact of ingestion of rice bran and shitake mushroom extract on lymphocyte function and cytokine production in healthy rats. *Journal of Dietary Supplements, 5*(1), 47-61. https://doi.org/10.1080/19390210802329196

Hoshino, Y., Hirashima, N., Nakanishi, M., & Furuno, T. (2010). Inhibition of degranulation and cytokine production in bone marrow-derived mast cells by hydrolyzed rice bran. *Inflammation Research, 59*(8), 615-625. https://doi.org/10.1007/s00011-010-0173-9

Kambayashi, H., & Endo, Y. (2002). Evaluation of the effects of asthma prevention and symptom reduction by enzymatically modified rice-bran foods in asthmatic model mice [Abstract] *Japanese Journal of Allergology, 51*(9/10), 957-957. https://doi.org/10.15036/arerugi.51.957_3

Kang, S. J., Yang, H. Y., Lee, S. J., Kim, J. H., Hwang, S. J., & Hong, S. G. (2022). Immunostimulatory effect of rice bran fermented by lentinus edodes mycelia on mouse macrophages and splenocytes. *Journal of the Korean Society of Food Science and Nutrition, 51*(8), 743-750. https://doi.org/10.3746/jkfn.2022.51.8.743

Kim, D. J., Choi, S. M., Kim, H. Y., Kim, J. H., Ryu, S. N., Han, S. J., & Hong, S. G. (2011a). Evaluation of biological activities of fermented rice bran from novel black colored rice cultivar SuperC3GHi. *Korean Journal of Crop Science, 56*(4), 420-426.

Kim, D. J., Ryu, S.-N., Han, S. J., Kim, H. Y., Kim, J. H., & Hong, S. G. (2011b). In vivo immunological activity in fermentation with black rice bran. *The Korean Journal of Food And Nutrition, 24*(3), 273-281. https://doi.org/10.9799/KSFAN.2011.24.3.273

Kim, H. Y., Han, J. T., Hong, S. G., Yang, S. B., Hwang, S. J., Shin, K. S., Suh, H. J., & Park, M. H. (2005). Enhancement of immunological activity in exo-biopolymer from submerged culture of Lentinus edodes with rice bran. *Natural Product Sciences, 11*(3), 183-187.

Kim, S. P., Lee, S. J., Nam, S. H., & Friedman, M. (2018). The composition of a bioprocessed shiitake (Lentinus edodes) mushroom mycelia and rice bran formulation and its antimicrobial effects against Salmonella enterica subsp. enterica serovar Typhimurium strain SL1344 in macrophage cells and in mice. *BMC Complementary and Alternative Medicine, 18*(1). https://doi.org/10.1186/s12906-018-2365-8

Kim, S. P., Park, S. O., Lee, S. J., Nam, S. H., & Friedman, M. (2013). A polysaccharide isolated from the liquid culture of Lentinus edodes (Shiitake) mushroom mycelia containing black rice bran protects mice against a Salmonella lipopolysaccharide-induced endotoxemia. *Journal of Agricultural and Food Chemistry, 61*(46), 10987-10994. https://doi.org/10.1021/jf403173k

Kim, S. P., Park, S. O., Lee, S. J., Nam, S. H., & Friedman, M. (2014). A polysaccharide isolated from the liquid culture of Lentinus edodes (Shiitake) mushroom mycelia containing black rice bran protects mice against salmonellosis through upregulation of the Th1 immune reaction. *Journal of Agricultural and Food Chemistry, 62*(11), 2384-2391. https://doi.org/10.1021/jf405223q

Miura, T., Chiba, M., Miyazaki, Y., Kato, Y., & Maeda, H. (2004/2013). Chemical structure of the component involved in immunoregulation. In *BioBran/MGN-3 (Rice Bran Arabinoxylan Coumpound): Basic and clinical application to integrative medicine* (2nd ed., pp. 14-22). BioBran Research Foundation. (Reprinted from a report of 2004 Annual Meeting of the Japanese society of Applied Glycoscience.)

Noaman, E., Badr El-Din, N. K., Bibars, M. A., Abou Mossallam, A. A., & Ghoneum, M. (2008). Antioxidant potential by arabinoxylan rice bran, MGN-3/biobran, represents a mechanism for its oncostatic effect against murine solid Ehrlich carcinoma. *Cancer Letters, 268*(2), 348-359. https://doi.org/10.1016/j.canlet.2008.04.012

Pérez-Martínez, A., Valentín, J., Fernández, L., Hernández-Jiménez, E., López-Collazo, E., Zerbes, P., Schwörer, E., Nuñéz, F., Martín, I. G., Sallis, H., Díaz, M. Á., Handgretinger, R., & Pfeiffer, M. M. (2015). Arabinoxylan rice bran (MGN-3/Biobran) enhances natural killer cell-mediated cytotoxicity against neuroblastoma in vitro and in vivo. *Cytotherapy, 17*(5), 601-612. https://doi.org/10.1016/j.jcyt.2014.11.001

Tazawa, K., Ichihashi, K., Fujii, T., Omura, K., Anazawa, M., & Maeda, H. (2003). The oral administration of the Hydrolysis Rice Bran (HRB) prevents a common cold syndrome in elderly people based on immunomodulatory function. *Journal of Traditional Medicines, 20*(3), 132-141.

Yu, K. W., Shin, K. S., Choi, Y. M., & Suh, H. J. (2004). Macrophage stimulating activity of exo-biopolymer from submerged culture of Lentinus edodes with rice bran. *Journal of Microbiology and Biotechnology, 14*(4), 658-664.

Zhu, X., Okubo, A., Igari, N., Ninomiya, K., & Egashira, Y. (2017). Modified rice bran hemicellulose inhibits vascular endothelial growth factor-induced angiogenesis in vitro via VEGFR2 and its downstream signaling pathways. *Bioscience of Microbiota, Food and Health, 36*(2), 45-53. https://doi.org/10.12938/bmfh.16-016
